# DNA damaging agents boost the transcription of endothelin A receptor in high-grade serous ovarian cancer

**DOI:** 10.1186/s13046-025-03607-0

**Published:** 2025-12-06

**Authors:** Celia Roman, Rosanna Sestito, Valentina Caprara, Andrea Sacconi, Giovanni Blandino, Anna Bagnato, Piera Tocci

**Affiliations:** 1 Preclinical Models and New Therapeutic Agents Unit, Istituto di Ricovero e Cura a Carattere Scientifico (IRCCS), Regina Elena National Cancer Institute, Rome, Italy; 2 Translational Oncology Research Unit, IRCCS, Regina Elena National Cancer Institute, Rome, Italy

**Keywords:** Ovarian cancer, ET_A_R, DNA damage response, PARPi, ATR

## Abstract

The antineoplastic agents, Poly ADP-ribose polymerase (PARP) inhibitors (PARPi) and platinum-based drugs, marked a paradigm shifting in high-grade serous ovarian cancer (HG-SOC) treatment. Nonetheless, the HG-SOC vulnerability to these agents is challenged by the hyper-activation of adaptive pro-survival pathways. In this study, we report that the endothelin A receptor (ET_A_R), whose overexpression correlated with an unfavourable HG-SOC patient outcomes, predicted a worst prognosis in the homologous recombination proficient subgroup of patients. ET_A_R levels increased upon treatment with DNA damaging agents, as the PARPi olaparib, or cisplatin in patient-derived (PD) HG-SOC cells and HG-SOC cell lines, regardless of their *BRCA1/2* mutational status. Mechanistically, these compounds, inducing the DNA Damage Response (DDR) signalling, including ATM and ATR, leveraged the STAT3-dependent transcriptional machinery to induce ET_A_R expression. The genetic and pharmacological inhibition of ET_A_R has the ability to potentiate the efficacy of the PARPi with DDR inhibitors (DDRi) combination, blocking the ET_A_R-mediated evasion from PARPi-induced apoptosis and DNA damage. Clinically relevant, in PD HG-SOC xenografts, the co-targeting of PARP, ATR, and ET_A_R effectively suppressed the olaparib-induced ET_A_R upregulation and enhanced PARPi sensitivity, preventing metastatic dissemination. These results explain a resilience mechanism in which ET_A_R confers a therapeutic vulnerability that may be leveraged for therapy with ATR and ET_A_R inhibitors in combination with PARPi, holding the promise of developing new therapeutic strategies less prone to drug resistance.

**Supplementary Information:**

The online version contains supplementary material available at 10.1186/s13046-025-03607-0.

## Background

High-grade serous ovarian carcinoma (HG-SOC) is depicted as one of the most lethal gynaecological malignancies, representing the main cause of cancer-related death in women [1, 2], with an overall 40% chance of survival [3]. Despite the impressive therapeutic success associated to the use of platinum-based chemotherapy and poly ADP-ribose polymerase inhibitors (PARPi), in the maintenance regimens, HG-SOC patients deal with a short-lived response to these treatments [4–6]. The inadequate efficacy of such therapies arises from multiple factors, including genetic mutations, restoration of the DNA repair, tumour microenvironment perturbation and activation of signalling routes that facilitate tumour escape from drug-induced apoptosis, strengthening the tumour cell survival rate [2, 7]. Deciphering the drivers of such suboptimal treatment efficacy, remains the greatest clinical challenge to find effective therapeutic option for patients with HG-SOC. Into the plethora of actionable molecular targets emerging as survival determinants has been recognized the endothelin-1 (ET-1) system, comprising two G protein coupled receptors (GPCR), the ETA receptor (ET_A_R) and the ETB receptor (ET_B_R), that get activated upon ET-1 binding [8]. Clinically relevant, the high expression of ET_A_R has been associated to dismal patient prognosis [9–12]. In this regard, it has been demonstrated that ET-1 turns on protection of HG-SOC cells against drug-induced apoptosis through Bcl-2-dependent mechanism [13]. Furthermore, the bidirectional communication with the stromal compartment, strengths HG-SOC ability to escape from anti-cancer treatments [14, 15]. Thus, targeting the ET-1/ET-1 receptors (ET-1R) axis may update the landscape of the therapeutic choice for HG-SOC patient poor responder to DNA damaging agents.

The dysregulation of signal transduction pathways associated to changes in receptor expression has been described to have a pivotal role in anti-cancer drug response [16]. In many human malignancies accumulating evidences illustrated how DNA damaging agents may induce a constitutive elevated expression of receptors associated with poor patient prognosis [17–21]. In this regard, it has been recently reported how olaparib, by activating the NFκB-mediated pathway, induces a constitutive elevated expression of the GPCR adenosine A2B receptor in HG-SOC, sustaining tumour cell growth, migration and olaparib resistance [21]. Nevertheless, the signalling network activated by DNA damaging agents that lead to the upregulation of a repertoire of GPCR, whose inhibition might contribute to the achievement of a better PARPi response, is still partially elucidated.

Although PARPi have already revolutionised the management of HG-SOC, this treatment often results in incomplete tumour elimination, ultimately leading to therapy failure [4, 5]. Future directions have been focused on combining PARPi with inhibitors of alternative DNA repair pathways, such as those mediated by Ataxia Telangiectasia Mutated (ATM) and Ataxia Telangiectasia and Rad3-Related Protein (ATR), as a powerful strategy to enhance drug sensitivity to conventional therapeutic interventions [22, 23]. Identifying the pathways necessary for cancer cells to survive under the pressure of PARPi/platinum therapy would be of high clinical significance to increase drug response, preventing the appearance of recurrent disease.

In this scenario, our study contributes to advance the understanding of the molecular mechanisms that enable HG-SOC to evade DNA damage agent-based treatments. In detail, exploiting patient-derived (PD) cells and other HG-SOC cells with different *BRCA1/2* mutational status, along with PD xenograft (PDX) models of HG-SOC, we identify a signalling machinery activated by cytotoxic drugs, including olaparib and cisplatin, that, via DNA damage response (DDR) pathway, promotes ET_A_R overexpression, endorsing an ET_A_R-driven adaptive pro-survival signalling network. Leveraging the combination of PARPi + DDRi with ET_A_R targeting compounds, potentiating HG-SOC sensitivity to PARPi and holding the potential to disrupt the ET_A_R-mediated cell death escape, offers a hopeful therapeutic avenue to prevent the emergence of unresponsiveness to PARPi in HG-SOC patients exhibiting high ET_A_R expression.

## Methods

### Kaplan-Meier survival analyses

Normalized gene expression of HG-SOC patients was obtained from Broad Institute The Cancer Genome Atlas (TCGA) Genome Data Analysis Center (2016): TCGA data from Broad GDAC Firehose 2016_01_28 run. Broad Institute of MIT and Harvard. Dataset 10.7908/C11G0KM9. Progression-free survival (PFS) and overall survival (OS) were evaluated through a Kaplan–Meier analysis, with differences between survival curves assessed using the log-rank test. Hazard ratios were estimated with Cox proportional hazards regression. Patients were stratified into high and low gene expression groups based on positive versus negative z-scores, as well as according to homologous recombination (HR) deficiency (HRD) status. HRD scores for ovarian cancer patient were obtained from UCSC Xena (https://xenabrowser.net/).

### Cell culture and treatments

PD HG-SOC primary cells, as well as Kuramochi and OVCAR8 HG-SOC cell lines, were used in this study. PD HG-SOC were isolated and characterized as previously described, according to a protocol for ascitic sample collection [11]. The study protocol and clinical information were approved by the Institutional Review Board from Regina Elena, requiring the patient’s written informed consent. Kuramochi cells were obtained from a Japanese Collection of Research Bioresources Cell Bank. OVCAR8 cells were a kindly gift from Dr G. Damia (Laboratory of Gynecological Preclinical Oncology, Istituto di Ricerche Farmacologiche Mario Negri IRCCS, Milan, Italy). Cells were cultured in RPMI 1640 (Gibco, Thermo Fisher Scientific, USA) containing 1% penicillin-streptomycin and 10% fetal bovine serum in a cell culture incubator with a humidified atmosphere (5% CO2 at 37 °C). Cells were tested for mycoplasma contamination in a regular basis and were validated by short tandem repeat profiling. Before each experiment, cells were incubated in serum-free medium for 24 hours (h). Olaparib (AZD2281, cat. no. S1060, Selleckchem, United Kingdom) was added for 24–48 h at 5 or 10 µM. The PD HG-SOC olaparib-unresponsive clones (clone #1 and #2) were obtained by exposure to cyclically and gradually increasing concentrations of olaparib (from 1,75 to 160 µM). Primarily used doses of olaparib were based on an IC50 value of olaparib determined from a dose–response curve over 5 days in the parental cell line. Cisplatin (cat. no. 11734609-03, Sandoz, Switzerland) was added for 24–48 h at 5 or 10 µM. ATR kinase inhibitor (AZD6738, Ceralasertib, cat. no. HY-19323, MedChemExpress, USA) was added for 48 h at 2,5 µM. ATM kinase inhibitor (KU-55933, cat. no. S1092, Selleckchem, United Kingdom) was added for 48 h at 5 µM. Stattic (cat. no. S7024, Selleckchem) was added for 48 h at 10 µM. Macitentan (ACT-064992, Actelion Pharmaceuticals, Ltd., Switzerland), a dual ET_A_/ET_B_ receptor antagonist, was added for 48 h at 1 µM. The cyclic pentapeptide BQ-123 (cat. no. B150, Sigma-Aldrich) and Zibotentan (ZD4054, cat. no. SML1550, Sigma-Aldrich), two selective ET_A_ receptor antagonists, were added for 48 h at 1 µM. For UV irradiation, the cells were incubated in medium without phenol-red and exposed to UV-B irradiation (100 mJ/cm2) in the Bio-Sun irradiation apparatus (Vilber, France) following manufacturer’s instructions. Control cells were treated identically but without UV exposure. Subsequently, cells were cultured for 48 h with fresh RPMI 1640 medium in the cell culture incubator.

HR status of PD HG-SOC cells was profiled using Next Generation Sequencing (NGS). The library preparation was performed with 10 ng of genomic DNA, and the chip was automatically loaded on the Ion Chef System (Thermo Fisher Scientific). The sequencing run was carried out with Ion Torrent GeneStudio™ S5 System and the Oncomine™ Tumor Specific BRCA Expanded Panel Assay (Thermo Fisher Scientific). Sequences were aligned to the hg19 reference genome, and analysis was performed using Ion Torrent Reporter™ Software 5.18 (Thermo Fisher Scientific) with the Oncomine™ Extended 5.18v2 filter chain.

### RNA interference

For transient knockdown, PD HG-SOC and Kuramochi cells were transfected for 72 h with Dharmacon ON-TARGETplus siRNA oligonucleotides (Dharmacon, Horizon Discovery Ltd, USA) specific for STAT3 (si-STAT3, L-003544-00−0050), EDNRA (si-ET_A_R, L-005485-00−0050) or ATR (si-ATR, L-003202-00−0020) used at a final concentration of 100 nM. In parallel, a non-targeting Control Pool siRNA was used as negative control (si-CTR, D-001810-10−50). Lipofectamine RNAiMAX reagent (cat. no. 13778075, Thermo Fisher Scientific) was used as transfection reagent according to the manufacturer’s protocol.

### Western blotting

Lysis of cells was carried using RIPA buffer (50mM Tris-HCl pH 7.4, 250mM NaCl, 1% Triton X-100, 1% sodium deoxycholate and 0.1% SDS) complemented with a mixture of protease and phosphatase inhibitors. Total cell lysates were subjected to SDS-PAGE. Denatured samples were loaded onto a gel of polyacrylamide and transferred by using Trans-Blot transfer pack (Bio-Rad, USA). The membranes were blocked in EveryBlot Blocking Buffer (Bio-Rad). The incubation with primary antibodies (ab) was done in TBST (TRIS-buffered saline with 0.1% Tween 20) with 5% BSA, overnight at 4 °C. After subsequent washing steps, membranes were incubated with the corresponding secondary ab. The filters were developed by using the enhanced chemiluminescence detection substrate (Bio-Rad) in the Chemi-Doc Imaging System (Bio-Rad). The following primary ab were adopted: anti-ET_A_R (1:5000, cat. no. PA3-065, Invitrogen); anti-γH2A.X (phospho-gamma-H2A.X Ser-139) (1:1000, cat. no. 2577, Cell Signalling); anti-pATR (Ser428) (1:1000, cat. no. 2853, Cell Signalling); anti-ATR (1:1000; cat. no. 2790; Cell Signalling); anti-pATM (Ser1981) (D25E5) (1:1000, cat. no. 13050, Cell Signalling); anti-ATM (D2E2) (1:1000, cat. no. 2873, Cell Signalling); anti-pCHK1 (Ser345) (133D3) (1:1000, cat. no. 2348, Cell Signalling); anti-CHK1 (2G1D5) (1:1000, cat. no. 2360, Cell Signalling); anti-pCHK2 (T68) (C13C1) (1:1000, cat. no. 2197, Cell Signalling); anti-CHK2 (1:1000, cat. no. 2662, Cell Signalling); anti-pSTAT3 (Y705) (D3A7) (1:1000; cat. no. 9145, Cell Signalling); anti-STAT3 (D3Z2G) (1:1000, cat. no. 12640, Cell Signalling); anti-caspase 3 (D3R6Y) (1:1000, cat. no. 142200, Cell Signalling); anti-cleaved-caspase 3 (D175) (1:1000, cat. no. 9661, Cell Signalling); anti-cleaved-PARP (Asp 214) (1:1000, cat. no. 9541, Cell Signalling); anti-tubulin (DM1A) (1:200, cat. no. sc-32293, Santa Cruz Biotechnology) and anti-β-actin (AC-15) (1:5000, cat. no. 1978, Sigma-Aldrich). The densitometric analyses were done using Image J program.

### Quantitative RT-PCR

Total RNA was extracted using the Trizol reagent (Thermo Fisher Scientific) according to the manufacturer’s protocol. Nanodrop 1000 spectrophotometer (Thermo Fisher Scientific) was used to check the RNA concentration and purity. RNA reverse transcription was done using the Wonder RT cDNA Synthesis kit (Euroclone, Italy). Quantitative reverse transcription polymerase chain reaction (qRT-PCR) was performed using Luna Universal qPCR Master Mix (M3003X, New England Biolabs) on QuantStudio 6-Flex (Thermo Fisher Scientific), according to the manufacturer’s instructions. The mRNA expression levels were obtained by normalizing to cyclophilin-A mRNA expression. Final data analysis was achieved by using 2-ΔΔCt method. The primers used for qRT-PCR are listed below:

- EDNRA Forward: 5′-GGGATCACCGTCCTCAACCT-3′;
- EDNRA Reverse: 5′-CAGGAATGGCCAGGATAAAGG-3′.
- BCL-2 Forward: 5′-CTGCACCTGACGCCCTTCACC-3′;
- BCL-2 Reverse: 5′-CACATGACCCCACCGAACTCAAAGA-3′.
- BCL-XL Forward: 5′-CTGCACCTGACGCCCTTCACC-3′;
- BCL-XL Reverse: 5′-AACCAGCGGTTGAAGCGTT-3′.
- Cyclophilin-A Forward: 5′-TTCATCTGCACTG CCAAGAC-3′;
- cyclophilin-A Reverse: 5′-TCGAGTTGTCCACAGTCAGC-3′.

### Luciferase assay

To measure ET_A_R promoter activity, PD HG-SOC and Kuramochi cells were seeded in 12-well plates. The cell density was about 70–80% on the day of the transfection. The transcriptional activity of ET_A_R was studied by using 1000 ng of human ET_A_R luciferase reporter plasmid containing a 980 base pare (bp) sequence from the ET_A_R promoter (−825 bp/+155 bp) synthesized by Neo-Biotech (France). The transcriptional activity of STAT3, performed in PD HG-SOC or Kuramochi cells seeded in 12 well-plates, was studied by using 300 ng STAT3 luciferase reporter vector or a negative control reporter (STAT3 reporter kit, BPS Bioscience, #79730, San Diego, USA). Mutant ET_A_R promoter reporter plasmid, carrying deletions of all three STAT3 binding sites (−180/−480), was generated by using the QuikChange II Site-Directed Mutagenesis Kit (#200521, Agilent Technologies, USA) and the following primers: Fw: 5′-GAAGCAAACCACCTGGGGGCGGCAGCTTTG-3′; Rev: 5′-CAAAGCTGCCGCCCCCAGGTGGTTTGCTTC-3′. All cells were co-transfected with 250 ng of pCMV-β-galactosidase vector (Promega, USA). The transfection was carried by using Lipofectamine 2000 (cat. no. 11668019, Thermo Fisher Scientific), according to manufacturer’s instructions. The luciferase assay system (Promega) was used to measure the reporter activity that was later normalized to β-galactosidase activity.

### Immunofluorescence

Cells were fixed with formaldehyde (4%) and subsequently permeabilized and blocked as previously described [14]. The incubation of primary abs anti-ET_A_R (1:200, cat. no. PA3-065; Invitrogen), anti-γH2A.X (phospho-gamma-H2A.X Ser-139; 1:300, cat. no. 05–636, Millipore, MA, USA) or anti-pSTAT3 (Y705) (1:150; cat. no 9145 S, Cell Signalling) was done overnight at 4 °C. On the following day, goat anti-rabbit Alexa Fluor 488 (1:500, cat. no. A11008; Thermo Fisher Scientific) and goat anti-mouse Alexa Fluor 647 (1:200, cat. no. A11001, Thermo Fisher Scientific) were added for the detection of ET_A_R and gamma-H2A.X, respectively; goat anti-mouse Alexa Fluor 488 for gamma-H2A.X and goat anti-rabbit Alexa Fluor 488 for pSTAT3. DAPI (PureBlu DAPI nuclear staining dye, cat. no. 1351303, Bio-Rad) was used to stain the nuclei. The samples were visualized in a Leica DMIRE2 deconvolution microscope furnished with a Leica DFC 350FX camera (Leica, Germany) using a 63X magnification. For γH2A.X foci analysis, an average of 200 cells per condition was scored and examined in ImageJ. Cells containing ≥ 5 γH2A.X foci were defined as foci positive, and the percentage of positive cells was calculated as (number of foci-positive nuclei/total number of nuclei scored) x 100. For ET_A_R analysis and the number of γH2A.X foci, an average of 200 cells per condition was analysed. The mean fluorescence intensity for ET_A_R or the number of γH2A.X foci were normalized to the number of cells and quantified using ImageJ.

### Chromatin Immunoprecipitation (ChIP) assay

Cells were crosslinked with formaldehyde (1%) in PBS for 8 min at room temperature. Chromatin, from 7 × 10^6^ cells per condition, was sheared by sonication, centrifuged and further diluted in 50mM Tris pH 8.0, 0.5% NP-40, 0.2 M NaCl, 0.5mM EDTA. For the input, one-twentieth of the precleared chromatin was used. The precleared chromatin was incubated overnight on a rotating shaker with anti-pSTAT3 (D3A7; dilution 1:50; cat. no. 9145S, Cell Signalling), or anti-rabbit IgG Isotype Control (Thermo Fisher Scientific). PCR was performed with the co-immunoprecipitated DNA with the following primers:

- ET_A_R primers STAT3 binding site Forward: 5′-TACAAGGGACAAGATAGAAGCAA-3′;
- ET_A_R primers STAT3 binding site Reverse: 5′-AAAAAGAAAACTCCGGAGGAACA-3.

### Cell viability assay

PD HG-SOC or Kuramochi cells (6 × 10^3^), were seeded in 96 opaque-walled multiwell plates and treated or not with the following drugs for 48 h, ATRi, olaparib, macitentan, BQ123 or zibotentan. When specified the cells were previously transfected with si-CTR or si-ET_A_R before seeding. After that, 100 µl of CellTiter-Glo 2.0 Reagent (cat. no. G9241, Promega) was added to an equal volume of cell culture medium present in each well following manufacturer’s protocol. The luminescent signal, reflecting the total amount of ATP associated to the presence of metabolically active cells, was recorded in a luminometer.

### Invasion assay

Transwell invasion assay was performed by seeding PD HG-SOC or Kuramochi cells (5 × 10^4^) in the upper part of the Boyden Chamber (8 μm pore size; Greiner Bio-One) pre-coated with Cultrex Culture Matrix (cat. no. 3445-005−01; R&D Systems) and medium containing 10% FBS was added to the lower chamber. After 48 h of treatment with olaparib in combination or not with ATRi, invaded cells were fixed and stained with the DIP Quick Stain kit (cat. no. BS156, Dyaset, Italy). Pictures were taken with the ZOE Fluorescent Cell Imaging System (Bio-Rad) at 20X magnification and invaded cells were counted by using ImageJ program.

#### In silico transcription factor binding site analysis

Putative Transcription Factor (TF)-target regulations for ET_A_R were obtained from hTFtarget (http://bioinfo.life.hust.edu.cn/hTFtarget#!/). Enrichment analysis was performed using EnrichR tool (https://maayanlab.cloud/Enrichr/) with MSigDB_Hallmark gene set.

### RNA sequencing of patient-derived HG-SOC cells

RNA sequencing (RNA-Seq) was performed as previously reported [14]. Specifically, total RNA was isolated from the PD HG-SOC cells treated or not with macitentan (1 µM) for 24 h using the RNeasy Quick Start kit (Qiagen, Germany), according to manufacturer’s instructions. Gene Set Enrichment Analysis (GSEA) of signalling pathways from Hallmark and Kyoto Encyclopedia of Genes and Genomes (KEGG) gene sets was performed using the Java version of the software (gsea2–2.2.3.jar; software.broadinstitute.org/gsea/), with log2FC obtained from RNA-Seq contrasts and standard parameters. The data of the RNA-Seq have been uploaded to the Gene Expression Omnibus (GEO) database (https://www.ncbi.nlm.nih.gov/geo/), with accession number GSE196065.

### Patient-derived xenografts (PDX) studies

Procedures involving animals and their care were guided with the permission from the IRCCS Regina Elena Cancer Institute Animal Care and Use Committee and the Italian Ministry of Health (D.lgs 26/2014, authorization number 1083/2020PR, issued 5 November 2020 by Ministero della Salute). Female athymic nude-CD1 nu+/nu + mice of six- to eight-week-old (Envigo Laboratories, IN, USA) were housed under pathogen-free conditions and HG-SOC PDX models were generated as previously described [11]. Following seven days of latency, the mice were randomly split into control (CTR; vehicle) group, ATRi (ceralasertib; 50 mg/kg/oral daily) group, olaparib (50 mg/kg/oral daily) group, macitentan (MAC; 30 mg/kg/oral daily) group and the combination ATRi + olaparib; ATRi + macitentan or ATRi + olaparib + macitentan groups. Body weight and signs and symptoms of stress due to the progression of the disease were monitored during the study. Following four weeks of treatment, mice were euthanized by cervical dislocation and the intraperitoneal metastatic lesions were counted and taken for further analysis. Values represent the mean of the number of visible metastatic lesions ± SD of 8 mice in each condition. The drug combination effect was assessed by determining the coefficient of drug interaction (CDI) based on the Chou-Talalay method [24]. A CDI value of less than 1 indicates a synergistic effect.

### Statistical analyses

Data are expressed as mean and standard deviation (SD). With the exception of the in vivo studies, data points from graphs represent the mean of three independent experiments performed in triplicates for all the conditions described. For the statistical analyses, Student’s t-test was applied to compare two groups of independent samples and to evaluate the sample size of mice for each treatment group. The statistical evaluation of cell survival over time for each treatment concentration was carried out using a two-way ANOVA test. The p-values were indicated in the legend of each figure. A threshold *p* < 0.05 was considered statistically significant. The statistical analyses were conducted using GraphPad Prism v9.0.

## Results

### High ET_A_R expression and homologous recombination proficiency (HRP) define a poor prognosis subtype of HG-SOC

**Fig. 1:**
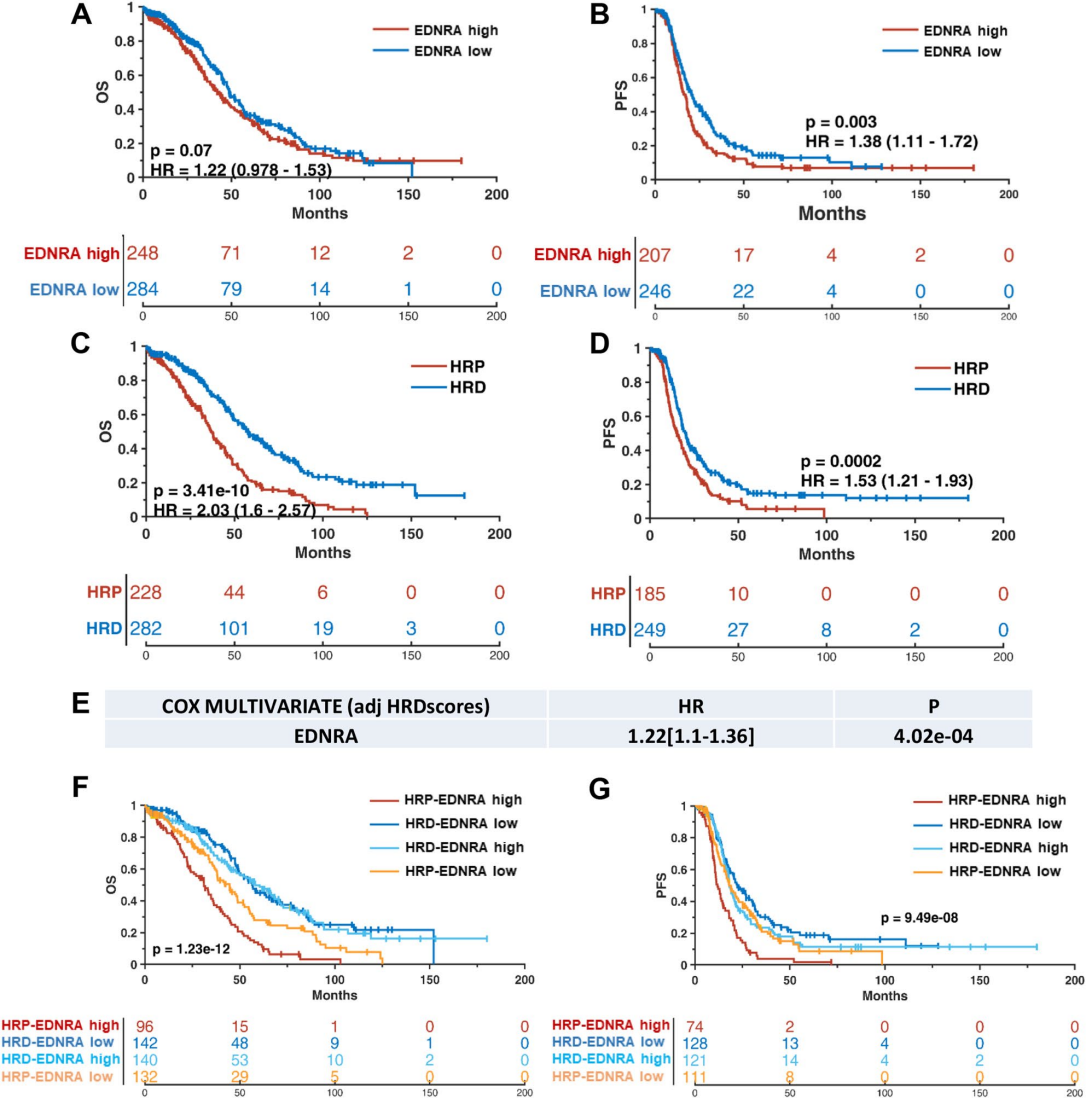
High ET_A_R expression and homologous recombination proficiency (HRP) define a poor prognosis subtype of HG-SOC patients. **A-D** Kaplan-Meier curves comparing overall survival (OS; **A**, **C**) and progression-free survival (PFS; **B**, **D**) generated by the analysis of gene expression data of high-grade serous ovarian cancer (HG-SOC) patients from the Cancer Genome Atlas (TCGA) dataset grouped according to the high/low EDNRA mRNA expression (**A**, **B**) or to the homologous recombination status (HRP) or homologous recombination deficiency (HRD); (**C**, **D**). **E** Multivariate Cox regression analysis adjusted to HRD status to evaluate the role of EDNRA gene as a predictor of poor survival [hazard ratio-HR = 1.22(1.1–1.36); p value = 4.02e-04]. **F**,** G** Kaplan-Meier analyses for OS (**F**) and PFS (**G**) of HG-SOC patients from TCGA grouped on the basis of both EDNRA mRNA expression and homologous recombination status

Gaining a deeper insight into biomarkers whose expression pattern can stratify HG-SOC patients as good or poor responders to therapy or according to their favourable or unfavourable prognosis, remains a major clinical challenge, especially for those patients deficient in the HR DNA repair pathways, initially sensitive to platinum/PARPi therapy [25]. In this respect, it has been reported that the HR status [26] and ET_A_R gene (EDNRA) overexpression [9–12] have been independently associated to a reduced life perspective and to a lower therapy responsiveness in HG-SOC patients. However, the prognostic value of their combined occurrence has not yet been defined. To address this gap, we investigated the potential interplay between the HR status and EDNRA mRNA expression, in relation to their combined impact on patient clinical outcomes, analysing a cohort of 587 HG-SOC patients from the TCGA dataset [27]. HG-SOC patients were first stratified for high and low ET_A_R expression levels, showing that the cohort of those highly expressing ET_A_R exhibited shorter OS and PFS compared to those with low ET_A_R expression (Fig. 1A, B). When patients were stratified by HR status, the HRP subgroup of patients demonstrated a shorter survival metric compared to the HRD subgroup (Fig. 1C, D). The multivariate Cox regression analysis suggested that HR and EDNRA were independent factors significantly impacting on HG-SOC patient’s prognosis (Fig. 1E). Remarkably, we observed that the combined presence of HRP and high ET_A_R identified a subset of patients with the poorest survival outcome across the entire cohort (Fig. 1F, G). These findings indicate ET_A_R as a valuable prognostic marker within the context of HRP, and emphasize the urgent need for new therapeutic interventions specifically tailored to target such particularly high-risk HG-SOC patient subgroup.

### DNA damaging agents induce the overexpression of endothelin A receptor

**Fig. 2:**
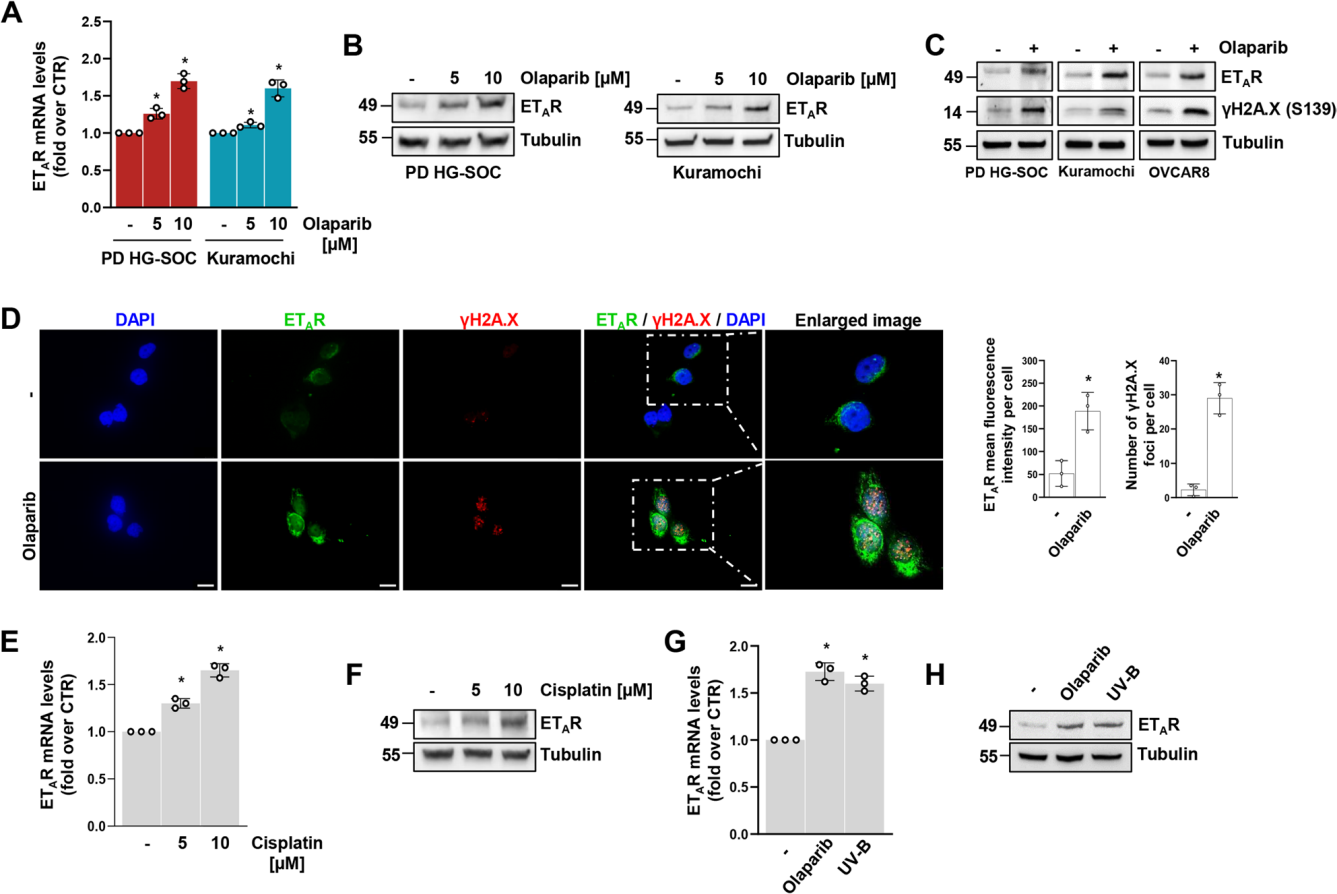
DNA damaging agents induce the overexpression of endothelin A receptor. **A** Quantitative RT-PCR (qRT-PCR) analysis of ET_A_R mRNA expression levels (fold over CTR) of PD HG-SOC and Kuramochi cells treated with 5 or 10 µM olaparib for 48 h (h). Data are presented as mean ± SD (*n* = 3, *p < 0,04 vs. non-treated cells). **B** Immunoblotting (IB) of ET_A_R protein expression levels of PD HG-SOC cells and Kuramochi cells treated as in *A*. Tubulin was used as loading control. **C** IB analysis assessing the ET_A_R and γH2A.X protein expression levels in PD HG-SOC, Kuramochi and OVCAR8 cells treated or not with 10 µM olaparib for 48 h. **D** Representative images from immunofluorescence (IF) analysis of ET_A_R and γH2A.X of Kuramochi cells treated as in *C*. Scale bar: 20 μm. Right graphs show the mean fluorescence intensity of ET_A_R immunostained Kuramochi cells and the number of γH2A.X foci per cell represented as mean ± SD. An average of 200 cells per condition was analysed (*n* = 3, *p < 0,005 vs. non-treated cells). **E** qRT-PCR analysis of ET_A_R mRNA expression levels (fold over CTR) of PD HG-SOC cells treated with 5 or 10 µM cisplatin for 48 h. Data are presented as mean ± SD (*n* = 3, *p < 0,01 vs. non-treated cells). **F** IB of ET_A_R protein expression levels of PD HG-SOC cells treated as in *E*. **G** qRT-PCR analysis of ET_A_R mRNA expression levels (fold over CTR) of PD HG-SOC cells treated with 10 µM olaparib or exposed to 100 mJ/cm^2^ UV-B irradiation. Data are presented as mean ± SD (*n* = 3, *p < 0,006 vs. non-treated cells). **H** IB of ET_A_R protein expression levels of PD HG-SOC cells stimulated as in *G*

The ET-1/ET_A_R axis is distinguished for its ability to activate autocrine/paracrine feed-forward loops that endow tumour cells with key survival attributes, sustaining HG-SOC progression [14, 15]. However, whether and how ET_A_R expression may be aberrantly perturbed by anti-cancer treatments is still unexplored. To monitor the expression of ET_A_R in response to treatment with the DNA damaging agents, we employed representative HG-SOC cells all harbouring mutant p53 but with different *BRCA1* and *BRCA2* mutational status, including PD HG-SOC primary cells, *BRCA1* and *BRCA2* wild-type, the HG-SOC cell lines Kuramochi and OVCAR8, harbouring *BRCA2* mutation (R2318) and *BRCA1* promoter hypermethylation, respectively (Supplementary Fig. S1A). In all the HG-SOC cells, we observed, independently of HR status, a dose-dependent increase of ET_A_R expression, at both mRNA and protein level, in response to treatment with olaparib, with a peak at 10 µM dosage (Fig. 2A, B), that occurs concurrently with DNA damage, as evidenced by the marked increase of γH2A.X expression levels (Fig. 2C). These observations were validated by an immunofluorescence analysis highlighting that, in response to olaparib treatment, HG-SOC cells were characterized by an enhancement in ET_A_R signal intensity that was concomitant with the accumulation of DNA damage, as demonstrated by the increase in the number of γH2A.X nuclear foci (Fig. 2D and Supplementary Fig. S1B). To substantiate these observations, we performed the same experiments upon cell treatment with another DNA damaging agent, cisplatin [28]. To an extent similar to olaparib, cisplatin, dose-dependently, induced ET_A_R upregulation (Fig. 2E, F). To further demonstrate the DNA damaging ability to enhance ET_A_R expression, we exposed PD HG-SOC cells to UV-B irradiation. In accordance with the effect produced by olaparib and cisplatin, UV-B irradiation enhanced the expression, at both mRNA and protein level, of ET_A_R (Fig. 2G, H). Altogether, these findings indicated an association between treatment-mediated DNA damage and the upregulation of ET_A_R in HG-SOC cells.

### Olaparib- or cisplatin-induced activation of the DDR cascade mediates ET_A_R upregulation

**Fig. 3:**
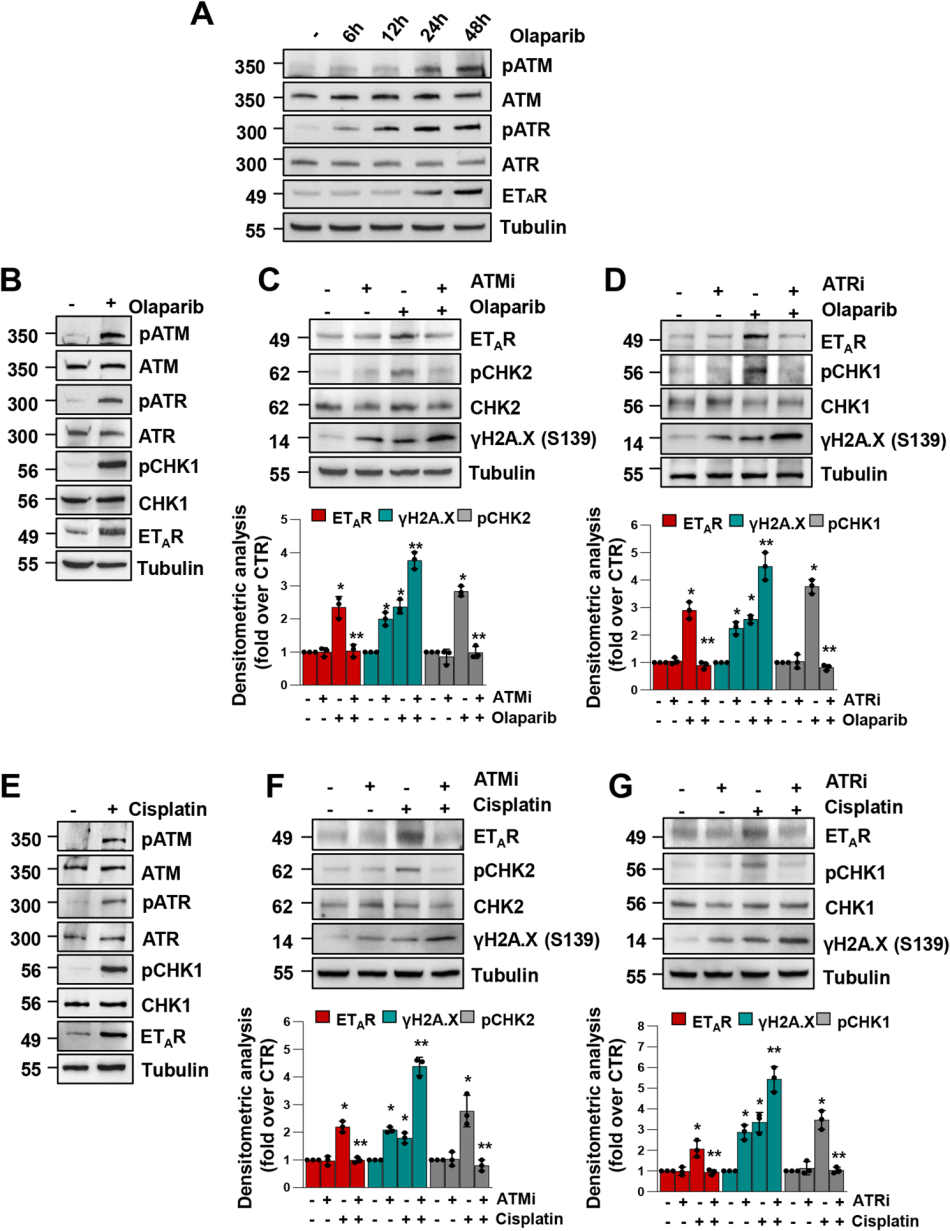
Olaparib- or cisplatin-induced activation of the DNA Damage Response cascade mediates ET_A_R upregulation. **A** IB of pATM, ATM, pATR, ATR and ET_A_R protein expression levels of PD HG-SOC cells treated or not with 10 µM olaparib for the indicated times. Tubulin was used as loading control. **B** IB of pATM, ATM, pATR, ATR, pCHK1, CHK1 and ET_A_R protein expression levels of PD HG-SOC cells treated or not with 10 µM olaparib for 48 h. **C** IB of ET_A_R, pCHK2, CHK2 and γH2A.X protein expression levels of PD HG-SOC cells treated with ATM inhibitor (ATMi, 5 µM) and/or olaparib (10 µM). Quantification of ET_A_R and γH2A.X protein expression normalized to tubulin, and pCHK2 normalized to total CHK2 and tubulin. All data are presented as mean ± SD expressed as fold induction (*n* = 3, *p < 0,02 vs. non-treated cells; **p < 0,008 vs. olaparib-treated cells). **D** IB of ET_A_R, pCHK1, CHK1 and γH2A.X expression levels of PD HG-SOC cells treated with ATR inhibitor (ATRi, 2,5 µM) and/or olaparib (10 µM). Quantification of ET_A_R, γH2A.X protein expression normalized to tubulin, and pCHK1 normalized to total CHK1 and tubulin (*n* = 3, *p < 0,02 vs. non-treated cells; **p < 0,02 vs. olaparib-treated cells). **E** IB of pATM, ATM, pATR, ATR, pCHK1, CHK1 and ET_A_R protein expression levels of PD HG-SOC cells treated or not with 10 µM cisplatin for 48 h. **F** IB of ET_A_R, pCHK2, CHK2 and γH2A.X protein expression levels of PD HG-SOC cells treated with ATMi (5 µM) and/or cisplatin (10 µM). Quantification of ET_A_R, γH2A.X and pCHK2 protein expression performed as in *C* (*n* = 3, *p < 0,04 vs. non-treated cells; **p < 0,002 vs. cisplatin-treated cells). **G** IB of ET_A_R, pCHK1, CHK1 and γH2A.X protein expression levels of PD HG-SOC cells treated with ATRi (2,5 µM) and/or cisplatin (10 µM). Quantification of ET_A_R, γH2A.X and pCHK1 protein expression performed as in *D* (*n* = 3, *p < 0,05 vs. non-treated cells; **p < 0,04 vs. cisplatin-treated cells)

In order to assess the mechanistic link existing between the DNA damaging functions and the upregulation of ET_A_R, we examined the activation on key components of the DDR pathway upon treatment with olaparib or cisplatin. In detail, the activation of the DDR pathway implies diverse signal kinase cascades, ruled by two DNA damage sensors, ATM and ATR, that favour the phosphorylation of the two downstream kinases, the checkpoint kinases CHK2 and CHK1 [29]. In light of this, in PD HG-SOC, Kuramochi and OVCAR8 cells, we observed that the olaparib-mediated ET_A_R upregulation followed the kinetic of DDR activation with a peak at 48 h (Fig. 3A and Supplementary Fig. S2A), supporting the notion that ET_A_R induction takes place downstream of DDR signalling activation, and was conjointly detected with the increase of pATM, pATR and pCHK1 levels after maximal DDR activation (Fig. 3B and Supplementary Fig. S2B, C).

To confirm the involvement of the DDR pathway in the ET_A_R upregulation, we measured the expression of ET_A_R and of DDR players in cells treated with olaparib or an ATM inhibitor (ATMi, KU-55933) alone or in combination. We observed that the olaparib-induced ET_A_R overexpression was distinctly suppressed upon cell treatment with a combination of olaparib and ATMi, concurrently blocking the downstream protein kinase CHK2 phosphorylation (Fig. 3C and Supplementary Fig. S2D). Similarly, a specific ATR inhibitor (ATRi, ceralasertib), currently being tested in clinical trials that enrol patients with advanced solid tumours, including HG-SOC [5, 30], by preventing the ATR-mediated phosphorylation of CHK1, interfered with the olaparib-induced ET_A_R upregulation (Fig. 3D and Supplementary Fig. S2D). These data suggest that the use of DDR inhibitors together with olaparib, interferes with the DNA damage kinase activity occurring upstream of ET_A_R induction, while potentiating DNA damage, as evinced by the increase of γH2A.X expression (Fig. 3C, D and Supplementary Fig. S2D). In the same way, the cisplatin-mediated activation of the ATM/ATR pathway correlated with ET_A_R upregulation (Fig. 3E), while ATMi or ATRi, in combination with cisplatin, interfered with the cisplatin-mediated ET_A_R induction by blocking ATM and ATR signalling, respectively (Fig. 3F, G). Overall, our results demonstrated that PARPi and platinum-based therapy, through the DDR pathway activation, led to the ET_A_R upregulation and that ATR or ATM targeting compounds, impairing the DDR kinase activity, exhibited the ability to suppress such olaparib/cisplatin-induced upregulation, suggesting that PARPi + DDRi combination therapy may enhance response to DNA-damaging agents.

### DDR activation mediates transcriptional upregulation of ET_A_R

**Fig. 4:**
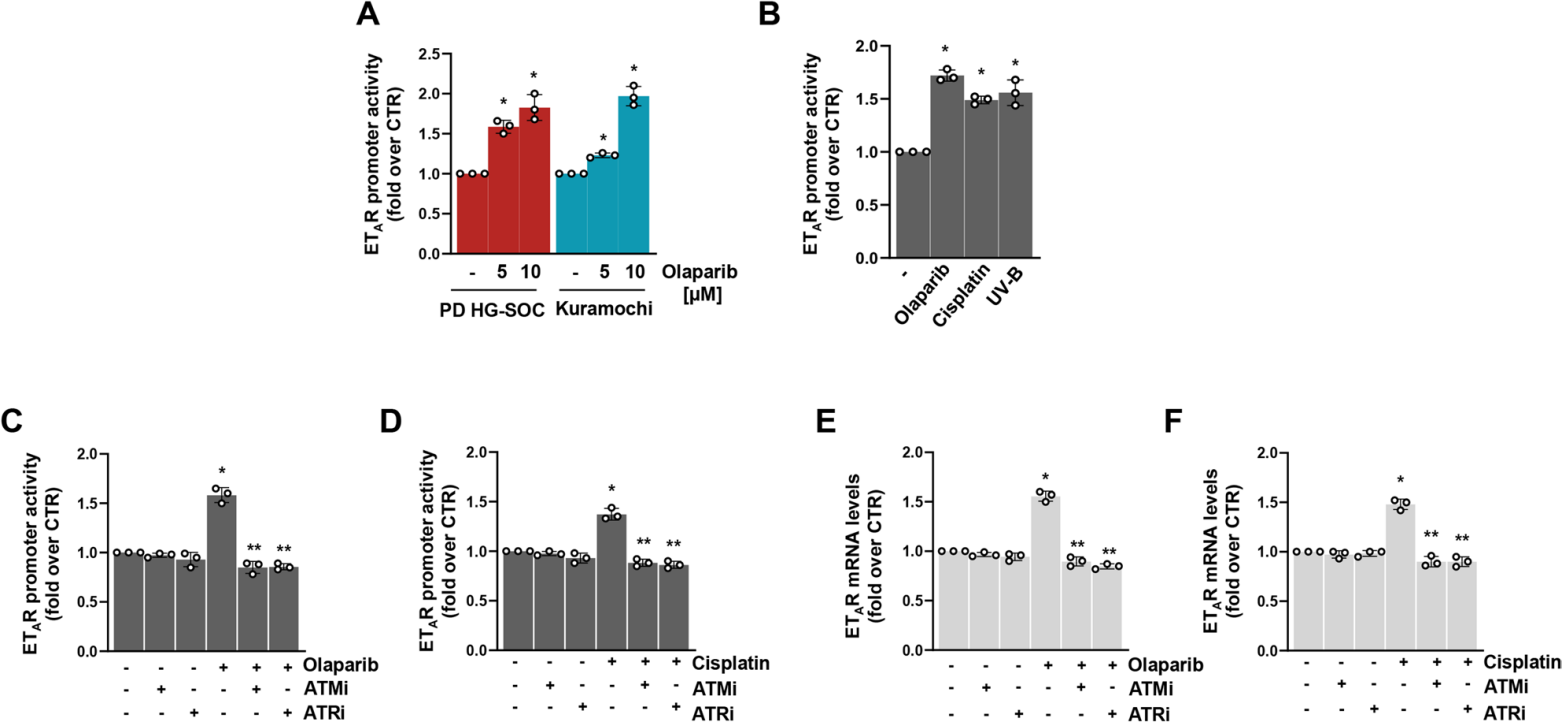
DDR activation mediates transcriptional upregulation of ET_A_R. **A** ET_A_R promoter activity (fold over CTR) of PD HG-SOC and Kuramochi cells treated with 5 or 10 µM olaparib. Data are presented as mean ± SD (*n* = 3, *p < 0,02 vs. non-treated cells). **B** ET_A_R promoter activity (fold over CTR) of PD HG-SOC cells treated with 10 µM olaparib or cisplatin or exposed to 100 mJ/cm^2^ UV-B irradiation. Data are presented as mean ± SD (*n* = 3, *p < 0,02 vs. non-treated cells). **C** ET_A_R promoter activity (fold over CTR) of PD HG-SOC cells treated with olaparib (10 µM) and/or ATMi (5 µM) or ATRi (2,5 µM). Data are presented as mean ± SD (*n* = 3, *p < 0,006 vs. non-treated cells; **p < 0,002 vs. olaparib-treated cells). **D** ET_A_R promoter activity (fold over CTR) of PD HG-SOC cells treated with cisplatin (10 µM) and/or ATMi (5 µM) or ATRi (2,5 µM). Data are presented as mean ± SD (*n* = 3, *p < 0,009 vs. non-treated cells; **p < 0,0008 vs. cisplatin-treated cells). **E** qRT-PCR analysis of ET_A_R mRNA expression levels (fold over CTR) of PD HG-SOC cells treated as in *C*. Data are presented as mean ± SD (*n* = 3, *p < 0,003 vs. non-treated cells; **p < 0,0004 vs. olaparib-treated cells). **F** qRT-PCR analysis of ET_A_R mRNA expression levels (fold over CTR) of PD HG-SOC cells treated as in *D*. Data are presented as mean ± SD (*n* = 3, *p < 0,005 vs. non-treated cells; **p < 0,0003 vs. cisplatin-treated cells)

The observed upregulation of ET_A_R at mRNA level following HG-SOC cell exposure to DNA damaging agents raises the question of whether such compounds could turn on the ET_A_R promoter activity, indicating that olaparib and cisplatin might shape ET_A_R expression by controlling its transcription. To tackle this aspect, we analysed the transcriptional activity of the ET_A_R promoter by employing a luciferase reporter system, revealing that treatment with olaparib resulted in a concentration-dependent upregulation of ET_A_R promoter function in PD HG-SOC and HG-SOC cells, reaching maximal induction at 10 µM (Fig. 4A). This effect was comparable to that one produced by cisplatin, as well as, upon cell exposure to UV-B irradiation (Fig. 4B), confirming that the ET_A_R promoter hyper-activation was the direct consequence of the DNA damaging agents. Further, both ATMi and ATRi, when combined with olaparib or cisplatin, were able to block both the olaparib- and cisplatin-induced ET_A_R promoter activity (Fig. 4C, D and Supplementary Fig. S2E), along with the ET_A_R mRNA level enhancement (Fig. 4E, F). Altogether, our results provide evidences on how the DNA damaging agents-dependent induction of ET_A_R occurs via a transcriptional mechanism.

### STAT3 transcriptional activity is required for the DNA damaging agents-induced ET_A_R upregulation

**Fig. 5:**
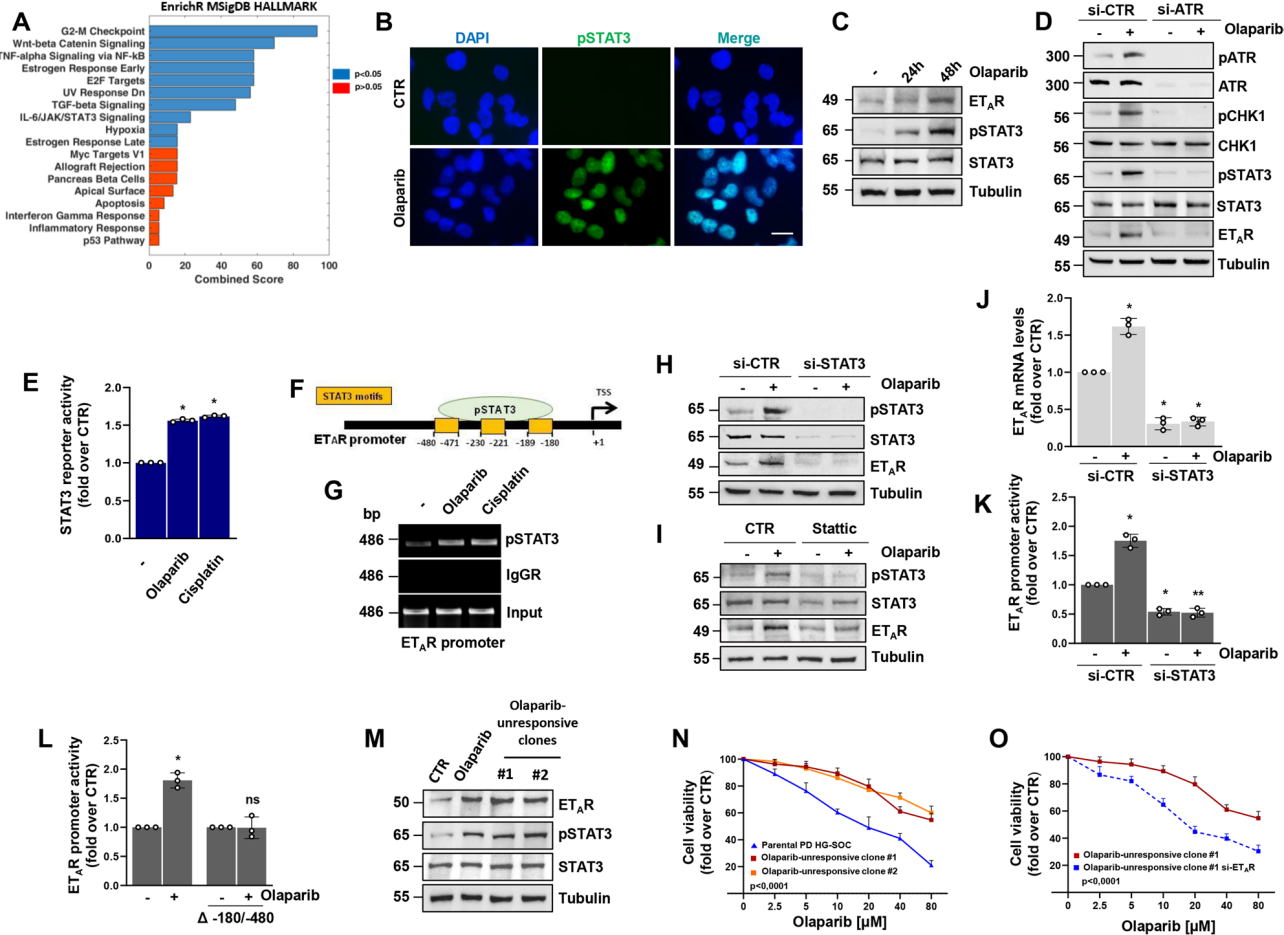
STAT3 transcriptional activity is required for the DNA damaging agents-induced ET_A_R upregulation. **A** Gene set enrichment analysis using the MSigDB_Hallmark gene set library. Significantly enriched pathways are coloured in blue (*p* < 0.05). **B** Representative images from IF analysis of pSTAT3 in PD HG-SOC cells treated or not with 10 µM olaparib for 48 h. Nuclei are stained in blue (DAPI), scale bar: 20 μm. **C** IB of ET_A_R, pSTAT3 and STAT3 protein expression levels from total extracts of PD HG-SOC cells treated with olaparib for 24–48 h. Tubulin was used as loading control. **D** IB of pATR, ATR, pCHK1, CHK1, pSTAT3, STAT3 and ET_A_R protein expression levels of PD HG-SOC cells transfected with si-CTR or si-ATR for 72 h and treated or not with olaparib for 48 h. **E** Luciferase activity (fold over CTR) detected in PD HG-SOC cells transfected with a STAT3 reporter vector and treated or not with 10 µM olaparib or cisplatin for 48 h. Data are presented as mean ± SD (*n* = 3, *p < 0,0005 vs. non-treated cells). **F** Representation of pSTAT3 canonical binding motifs in the ET_A_R promoter. **G** The recruitment of STAT3 in the ET_A_R promoter region was analyzed by ChIP assay followed by PCR in PD HG-SOC cells stimulated or not with olaparib or cisplatin for 24 h. Anti-IgG Rabbit ab (IgGR) was used as a control. **H** IB of pSTAT3, STAT3 and ET_A_R protein expression levels of PD HG-SOC cells transfected with si-CTR or si-STAT3 for 72 h and treated or not with olaparib for 48 h. **I** IB of pSTAT3, STAT3 and ET_A_R protein expression levels of PD HG-SOC cells treated with stattic (10 µM) and/or olaparib (10 µM) for 48 h. **J** qRT-PCR analysis of ET_A_R mRNA expression levels of PD HG-SOC cells transfected and treated as in *H*. Data are presented as mean ± SD (*n* = 3, *p < 0,01 vs. si-CTR non-treated cells; **p < 0,0004 vs. olaparib-treated cells). **K** ET_A_R promoter activity (fold over CTR) of PD HG-SOC cells transfected with si-CTR or si-STAT3 and with the ET_A_R promoter reporter plasmid and treated or not with olaparib for 48 h. Data are presented as mean ± SD (*n* = 3, *p < 0,08 vs. si-CTR non-treated cells; **p < 0,0003 vs. olaparib-treated cells). **L** ET_A_R promoter activity (fold over CTR) of PD HG-SOC cells transfected with the ET_A_R promoter reporter plasmid or its mutant lacking STAT3 binding sites (Δ−180/−480) and treated or not with olaparib for 48 h (*n* = 3, *p < 0,009 vs. non-treated cells transfected with the ET_A_R promoter reporter plasmid; n.s.= no significance vs. non-treated cells transfected with the mutant ET_A_R promoter reporter plasmid (Δ−180/−480)). **M** IB of pSTAT3, STAT3 and ET_A_R protein expression levels of the parental PD HG-SOC cells or the olaparib-unresponsive PD HG-SOC clones #1 and #2 treated or not with olaparib (10 µM) for 48 h. **N** Cell viability assay in parental PD HG-SOC cells and olaparib-unresponsive clones #1 and #2 treated with increasing doses of olaparib for 48 h. Relative luminescence units were used as an indicator of viability and displayed as mean ± SD, expressed as fold over CTR (*n* = 3, *p* < 0,0001 vs. non-treated parental PD HG-SOC cells). **O** Olaparib-unresponsive cells transfected with si-CTR or si-ET_A_R for 72 h and treated with increasing doses of olaparib for 48 h (*n* = 3, *p* < 0,0001 vs. si-CTR non-treated cells)

In view of the increase in the ET_A_R gene expression and ET_A_R promoter activity observed upon cell treatment with DNA damaging agents, our following goal was to identify which TF might be responsible of ET_A_R upregulation in response to olaparib or cisplatin in HG-SOC. In an effort to uncover such TF, we performed an in-silico analysis that revealed the top TF that could potentially bind their target regions on the ET_A_R promoter (Additional file 2 and 3), unveiling that one of the statistically significant (light blue bars, *p* < 0.05) was the IL-6/JAK/STAT3 signalling pathway, that might be implicated in the transcriptional regulation of ET_A_R (Fig. 5A). These data, along with recent findings highlighting the involvement of STAT3 in ET_A_R regulation [31], encouraged us to explore whether STAT3 could be in charge of the upregulated ET_A_R transcription in response to olaparib and cisplatin treatment. First, we examined that in HG-SOC cells olaparib treatment increased the phosphorylated (pSTAT3 Tyr705) and active form of STAT3 (Fig. 5B) along with ET_A_R expression (Fig. 5C and Supplementary Fig. S3A). In the attempt to understand how the blockade of the DDR impacts on STAT3 activity, we performed silencing experiments observing that the depletion of ATR, triggered STAT3 downregulation at both baseline level an upon olaparib treatment, consequently restraining the capability of STAT3 to boost the transcription of ET_A_R (Fig. 5D and Supplementary Fig. S3B). Moreover, we measured the STAT3 transcriptional activity by performing a reporter luciferase assay, evidencing an enhancement of STAT3 transcriptional functionality following olaparib or cisplatin treatments (Fig. 5E and Supplementary Fig. S3C). Next, we performed a ChIP analysis to assess the recruitment of pSTAT3 on the ET_A_R promoter region that possesses three canonical binding sites for STAT3 (Fig. 5F), revealing that olaparib or cisplatin treatment induced the recruitment of pSTAT3 on ET_A_R promoter (Fig. 5G), uncovering how these drugs stimulate the STAT3-driven transcriptional activation of ET_A_R. With the aim to prove the direct association between STAT3 transcriptional activation and ET_A_R expression, we depleted HG-SOC cells for STAT3, observing that its silencing abrogated ET_A_R protein and mRNA expression at both baseline level and after olaparib or cisplatin treatment (Fig. 5H, J and Supplementary Fig. S3D, E, H). Similarly, treatment with the STAT3 inhibitor stattic induced a down-modulation of ET_A_R following treatment with the DNA damaging agents (Fig. 5I and Supplementary Fig. S3F, G). In parallel, we observed that the promoter activity of ET_A_R, upregulated upon olaparib treatment, was impaired by STAT3 silencing (Fig. 5K and Supplementary Fig. S3I). Consistently with this observation, in HG-SOC cells transiently transfected with a mutant ET_A_R promoter construct, lacking the three canonical binding sites for STAT3 (Δ−180/−480), we found that olaparib lost the ability to upregulate the ET_A_R promoter luciferase activity, further substantiating the notion that in response to olaparib treatment pSTAT3 is recruited on ET_A_R promoter, leading to its induction (Fig. 5L). Moreover, we observed an upregulation of other STAT3 target genes, including BCL-XL and BCL2, following olaparib treatment that was abrogated upon STAT3 silencing, supporting the concept that cancer cells via STAT3 hijack the upregulation of survival effectors, as BCL-XL and BCL2 (Supplementary Fig. S3J). Taking together, these data suggest that the transcriptional ET_A_R upregulation, induced by DNA damage agents, requires STAT3 activation as an additional adaptive route to survive to the olaparib-induced apoptosis.

Mounting evidences indicated that ET_A_R overexpression in HG-SOC patients was strongly linked to a reduced life perspective and impaired therapy response to cisplatin, underscoring ET_A_R as a compelling biomarker and potential predictor of chemoresistance [9–12]. Considering the possibility that treatment with DNA damage agents could select resistant subclones expressing high levels of ET_A_R as a mechanism to adapt, we explored whether the overexpression of ET_A_R could actively support a cell state refractory to PARPi. To address this hypothesis, we generated two isogenic olaparib-unresponsive PD HG-SOC cell clones (clone #1 and #2). These clones displayed an increased ET_A_R expression and an enhanced STAT3 activation, with expression levels similar to those detected in olaparib-treated PD HG-SOC cells (Fig. 5M). Moreover, cell viability analyses revealed a greater ability of both clones to escape from olaparib-induced cell death compared to the parental PD HG-SOC cells, when exposed to different doses of olaparib (Fig. 5N), supporting the critical role of ET_A_R upregulation in conferring poor response to PARPi. ET_A_R silencing in the olaparib-unresponsive cells restored their sensitivity to this drug (Fig. 5O), underscoring how HG-SOC cells, under the selective pressure exerted by olaparib, might promote the upregulation of ET_A_R to gain a survival advantage. These results indicate that the hyperactivation of the ET_A_R-driven signalling allows cells to engage an adaptive mechanism to evade therapy and maintain a PARPi-unresponsive state.

### Combining olaparib with ET_A_R/ATR Inhibition potentiates DNA damage and apoptosis

**Fig. 6:**
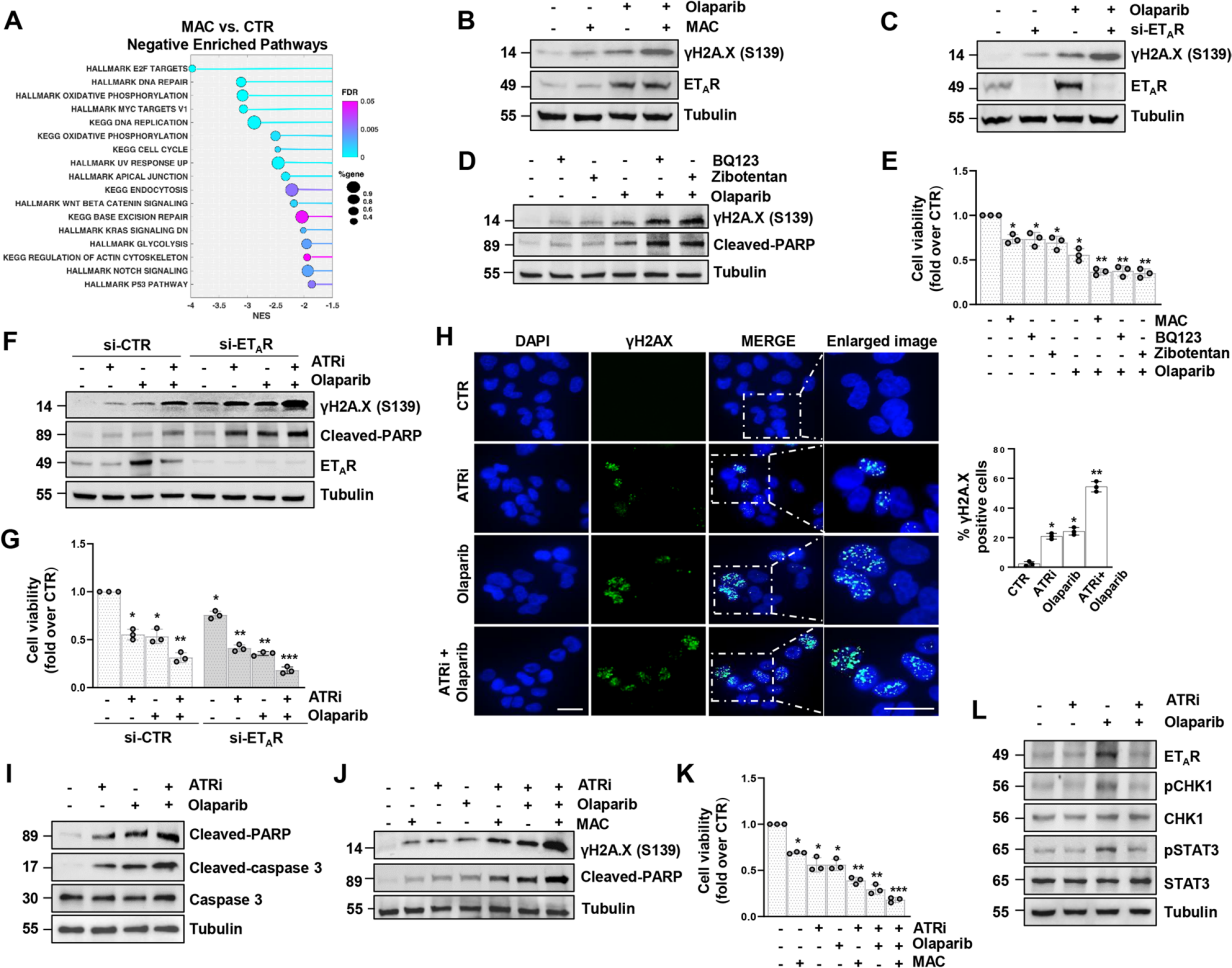
Combining olaparib with ET_A_R/ATR inhibition potentiates DNA damage and apoptosis. **A** Bubble chart of the top Hallmark and KEGG gene sets obtained from RNA-seq in PD HG-SOC cells upon macitentan (MAC, 1 µM) treatment for 24 h vs. control (CTR) cells. **B** IB of γH2A.X and ET_A_R protein expression levels of PD HG-SOC cells treated with macitentan (1 µM) and/or olaparib (10 µM) for 48 h. Tubulin was used as loading control. **C** PD HG-SOC cells transfected with si-CTR or si-ET_A_R for 72 h and treated or not with 10 µM olaparib evaluated for γH2A.X and ET_A_R protein expression by IB. **D** IB of γH2A.X and cleaved-PARP protein expression levels of PD HG-SOC cells treated or not with BQ123 (1 µM), zibotentan (1 µM) and/or olaparib (10 µM) for 48 h. **E** Cell viability in PD HG-SOC cells treated with macitentan, BQ123, zibotentan and olaparib alone or in combination. Relative luminescence units were used as an indicator of viability and displayed as mean ± SD, expressed as fold over CTR (*n* = 3; *p < 0,03 vs. non-treated cells; **p < 0,05 vs. olaparib-treated cells). **F** IB of γH2A.X, cleaved-PARP and ET_A_R protein expression levels of PD HG-SOC cells transfected with si-CTR or si-ET_A_R for 72 h and treated with ATRi and/or olaparib for 48 h. **G** Cell viability of PD HG-SOC cells treated as in *F* (*n* = 3; *p < 0,01 vs. si-CTR non-treated cells; **p < 0,05 vs. si-CTR ATRi- or olaparib-treated cells; ***p < 0,03 vs. si-CTR ATRi + olaparib-treated cells). **H** Representative images from IF analysis for γH2A.X in PD HG-SOC cells treated with ATRi and/or olaparib for 48 h. Nuclei are stained in blue (DAPI), scale bar: 20 μm. The right graph shows the percentage of γH2A.X-positive cells (cells having ≥ 5 foci), represented as mean ± SD. An average of 200 cells per condition was analysed (*n* = 3; *p < 0,0007 vs. non-treated cells; **p < 0,0006 vs. olaparib-treated cells). **I** IB of cleaved-PARP, cleaved-caspase 3 and total caspase 3 protein expression levels of PD HG-SOC cells treated with ATRi and/or olaparib. **J** IB of γH2A.X and cleaved-PARP protein expression levels of PD HG-SOC cells treated with ATRi, olaparib and/or macitentan for 48 h. **K** Cell viability in PD HG-SOC cells treated as in *J* (*n* = 3; *p < 0,02 vs. non-treated cells; **p < 0,05 vs. macitentan- or olaparib-treated cells; ***p < 0,05 vs. ATRi + olaparib-treated cells). **L** IB of ET_A_R, pSTAT3, STAT3, pCHK1 and CHK1 protein expression levels of PD HG-SOC cells treated with olaparib and/or ATRi

To deepen into the plethora of signalling networks downstream of ET_A_R, we conducted a RNA-Seq analysis in PD HG-SOC cells upon treatment with the ET-1R antagonist macitentan for 24 h. Interrogation of the differentially regulated genes revealed how in response to macitentan treatment vs. untreated cells (CTR), many signalling, including MYC and E2F targets, critically linked to therapy resistance [32], were interfered in macitentan-treated samples (Fig. 6A). This observation is in line with recent notions showing that in ovarian cancer the deregulation of the MYC signature may be exploited by cancer cells to survive to PARPi-based therapy [33, 34]. Beyond MYC, it has been reported in STAT3-silenced HG-SOC cells a downregulation of E2F target genes, known to be involved in cell cycle progression, DNA replication and DNA repair pathway [35], suggesting that shared STAT3 transcriptional program can be impaired by ET-1R blockade. To further extend the potential clinical impact of these observations, the inhibitory effect of macitentan were assessed in combination with olaparib on the expression of DNA damage markers. In particular, the pharmacological inhibition of ET_A_R combined with olaparib treatment enhanced cell sensitivity to PARPi, as proved by the increase of γH2A.X protein levels (Fig. 6B). Of note, comparable results were obtained upon ET_A_R depletion (Fig. 6C). In this regard, it has been reported that macitentan, thanks to its ability to simultaneously interfere the ET_A_R-driven signalling that support cancer cell activity and the ET_B_R-mediated route strongly active in stromal cells, may potentiate PARPi effects, when administered in combinatorial regimen in breast and ovarian cancer cells highly expressing the ET-1/ET-1R axis [14]. To strength the concept that the targeting of the ET_A_R-driven signalling may potentiate olaparib efficacy, we examine the effect in terms of apoptosis and DNA damage produced by two ET_A_R selective antagonists, BQ123 and zibotentan, administered alone or in combinatorial fashion with olaparib. To an extent comparable to that one achieved by macitentan, BQ123 and zibotentan, increased the expression of both γH2A.X and cleaved-PARP, as markers of DNA damage and apoptosis, respectively, and reduced PD HG-SOC cell viability (Fig. 6D, E). Such effects were more pronounced in cells treated with a combination of BQ123 + olaparib, or zibotentan + olaparib, with a response comparable to that one produced by the depletion of ET_A_R combined with olaparib treatment, or by macitentan + olaparib (Fig. 6D-G). Considering the reliance of ovarian cancer cells on the DDR pathway to repair DNA damage, DDR-targeting drugs are prime candidates to exploit synthetic lethality when used in combination with PARPi [36, 37]. In this view, we evaluated whether the inhibition of ATR would potentiate PARPi-mediated cytotoxicity. We observed that PARP and ATR dual inhibition resulted in a significantly higher increase of γH2A.X and cleaved-PARP (Fig. 6F and Supplementary Fig. S4A) and in the decline of cell viability (Fig. 6G and Supplementary Fig. S4B) when compared to olaparib treatment alone. Moreover, we observed a rise in γH2A.X positive cells (Fig. 6H) and a boost of apoptotic markers, as cleaved-PARP and cleaved-caspase 3 (Fig. 6I and Supplementary Fig. S4C). Additionally, compared to olaparib monotherapy, olaparib and ATRi combinatorial treatment hindered the invasiveness of HG-SOC cells (Supplementary Fig. S4D, E). Interestingly, the combination of olaparib and ATRi was further potentiated in cells silenced for ET_A_R (Fig. 6F, G and Supplementary Fig. S4A, B), or by using the combination of ATRi and olaparib with ET_A_R antagonists, either selective, as zibotentan, or not, as macitentan, showing an enhancement of DNA damage and apoptotic markers and inhibition of cell vitality (Fig. 6J, K and Supplementary Fig. S4F, G). Additionally, we observed that ATRi or ATMi, when combined with olaparib, suppressed the olaparib-triggered ET_A_R and STAT3 activation (Fig. 6L and Supplementary Fig. S4H). These data reveal that DDR inhibition in combination with PARPi, neutralizes the ET_A_R-mediated evasion from olaparib-induced apoptosis and DNA damage. The targeting of the ET_A_R receptor potentiates the anti-tumour effects of olaparib + ATRi combination, indicating the potential utility of macitentan as a componenent of combination therapies to sensitize cells to PARPi therapy.

### The combination of olaparib with macitentan and ATRi sensitizes to olaparib and hinders metastatic colonization in a HG-SOC PDX model

**Fig. 7:**
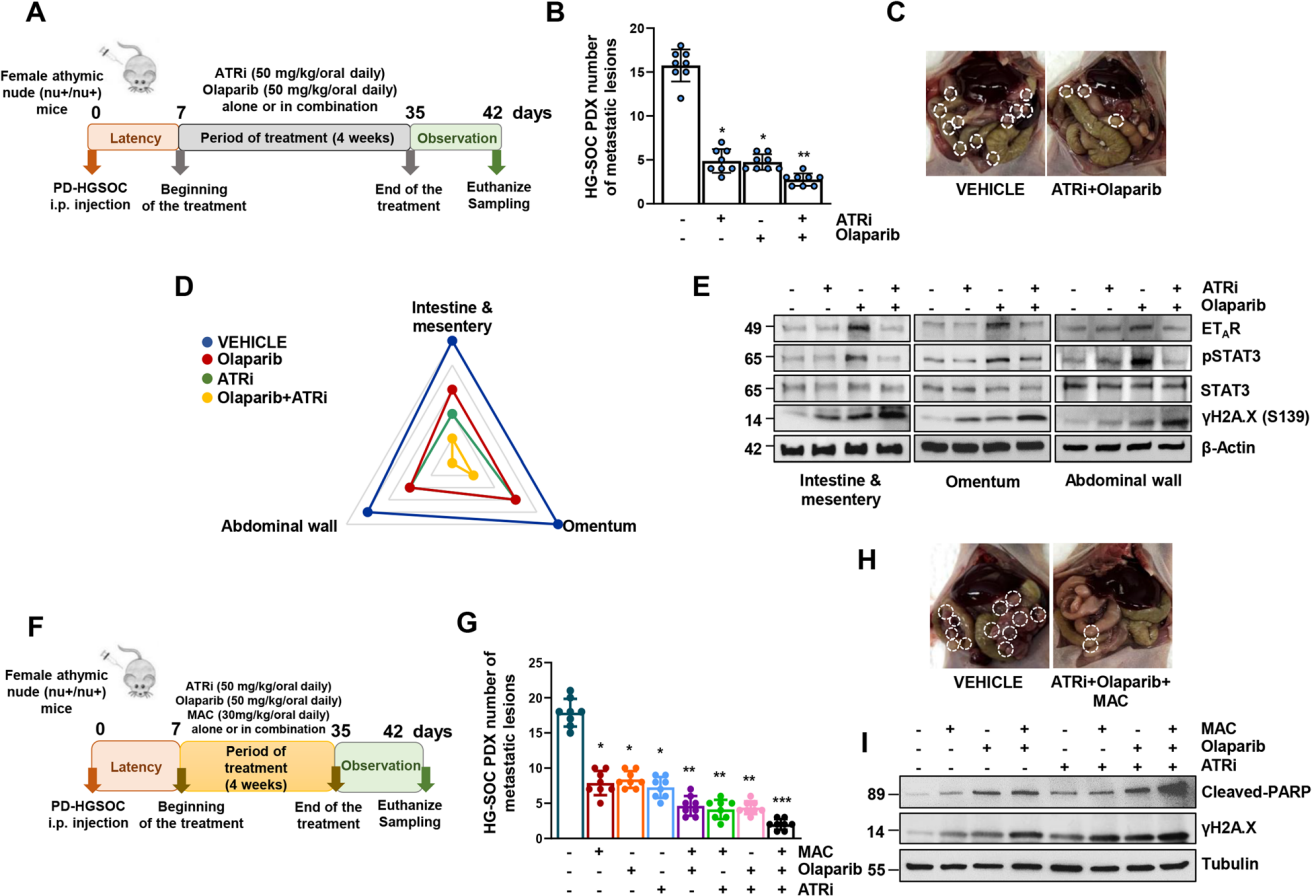
The combination of olaparib with macitentan and ATRi sensitizes to olaparib and hinders metastatic colonization in a HG-SOC PDX model. **A** Treatment schedule of patient-derived xenografts (PDX) HG-SOC treated with vehicle (CTR) or the PARPi (olaparib, 50 mg/kg/oral daily) and the ATRi (ceralasertib, 50 mg/kg/oral daily) in monotherapy or in combination therapy for four weeks. **B** The number of metastatic lesions examined at the end of the treatment. Data are presented as mean ± SD (8 mice/per condition, *p < 0,0001 vs. vehicle-treated mice (CTR); **p < 0,0003 vs. olaparib-treated mice). **C** Representative images of intraperitoneal tumour nodules, indicated by white circles, from PDX models treated with vehicle or the co-therapy olaparib + ATRi as indicated in *A*. **D** Radar plot reporting the distribution of metastasis, as determined by microscopic analysis, in mice injected intraperitoneal as indicated in *A*. **E** IB of ET_A_R, pSTAT3, STAT3 and γH2A.X protein expression levels from different tumour lesions (intestine & mesentery, omentum and abdominal wall) of PDX treated as in *A*. β-actin was used as loading control. **F** Treatment schedule of PDX treated with vehicle (CTR), PARPi (olaparib, 50 mg/kg/oral daily), ATRi (ceralasertib, 50 mg/kg/oral daily) and macitentan (MAC, 30 mg/kg/oral daily) in monotherapy or in double or triple combination therapy for four weeks. **G** The number of metastatic lesions examined at the end of the treatment. Data are presented as mean ± SD (8 mice/per condition, *p < 0,0002 vs. vehicle-treated mice (CTR); **p < 0,0004 vs. macitentan- or olaparib-treated mice, ***p < 0,0002 vs. ATRi + olaparib-treated mice). **H** Representative images of intraperitoneal tumour nodules, indicated by white circles, from PDX models treated as in *F*. **I** IB of cleaved-PARP and γH2A.X protein expression levels from tumour lesions of PDX models treated as in *F*. Tubulin was used as loading control

To expand the potential clinical relevance of our in vitro observations, we explored the possibility that the inhibition of the DDR pathway, by using ATR targeting compounds, as the ATRi ceralasertib, in a combinatorial regimen with olaparib, might halt the DNA damaging agents-triggered ET_A_R induction, lowering the HG-SOC metastatic potential in vivo. To track the HG-SOC metastatization and to follow up the therapeutic outcome achieved by the monotherapies and/or the combination of olaparib + ATRi, we employed a previously established HG-SOC PDX model [11]. In such preclinical setting we observed that the olaparib (50 mg/kg/oral daily for 4 weeks) + ATRi (50 mg/kg/oral daily for 4 weeks) combinatorial treatment schedule, more than olaparib alone, synergistically reduced the number of the metastatic lesions (n° of metastases in the olaparib + ATRi = 2,75 ± 0,68 vs. n° of metastases in olaparib-treated mice = 4,75 ± 0,85; Fig. 7A-E). The drug combination was well tolerated, with no weight loss evinced in all treatment groups. As evidenced by the radar plot, a macroscopic observation of the metastatic lesion distribution revealed the presence of metastatic nodules at the intestine and mesentery, the omentum, and the abdominal wall (Fig. 7D). The analysis of protein extracts obtained from these metastatic sites underscored that all the metastatic spots displayed a comparable expression pattern of ET_A_R and pSTAT3, further validating our in vitro observations according to which the olaparib-triggered upregulation of ET_A_R and pSTAT3 was attenuated by olaparib + ATRi (Fig. 7E). Along with these results, we observed that this combination of drugs displayed the ability to potentiate DNA damage, as shown by the increase of the γH2A.X protein expression (Fig. 7E). Next, we assessed in vivo the therapeutic benefit produced by the triple combination of PARPi + ATRi + macitentan. This combination was well-tolerated with no signs of toxicity in the mice. As evidenced in Fig. 7F-I, the administration of the three drugs, in a synergistic fashion, enhancing apoptosis and DNA damage, reduced the HG-SOC metastatic course with a greater effect compared to that one produced by PARPi + ATRi (Fig. 7G, I), opening to a compelling perspective in which the combination of macitentan + PARPi and ATRi has a great translational implication to enhance the PARPi therapeutic efficacy, hindering the HG-SOC malignant progression, particularly for HG-SOC patients with high ET_A_R expression.

## Discussion

**Fig. 8:**
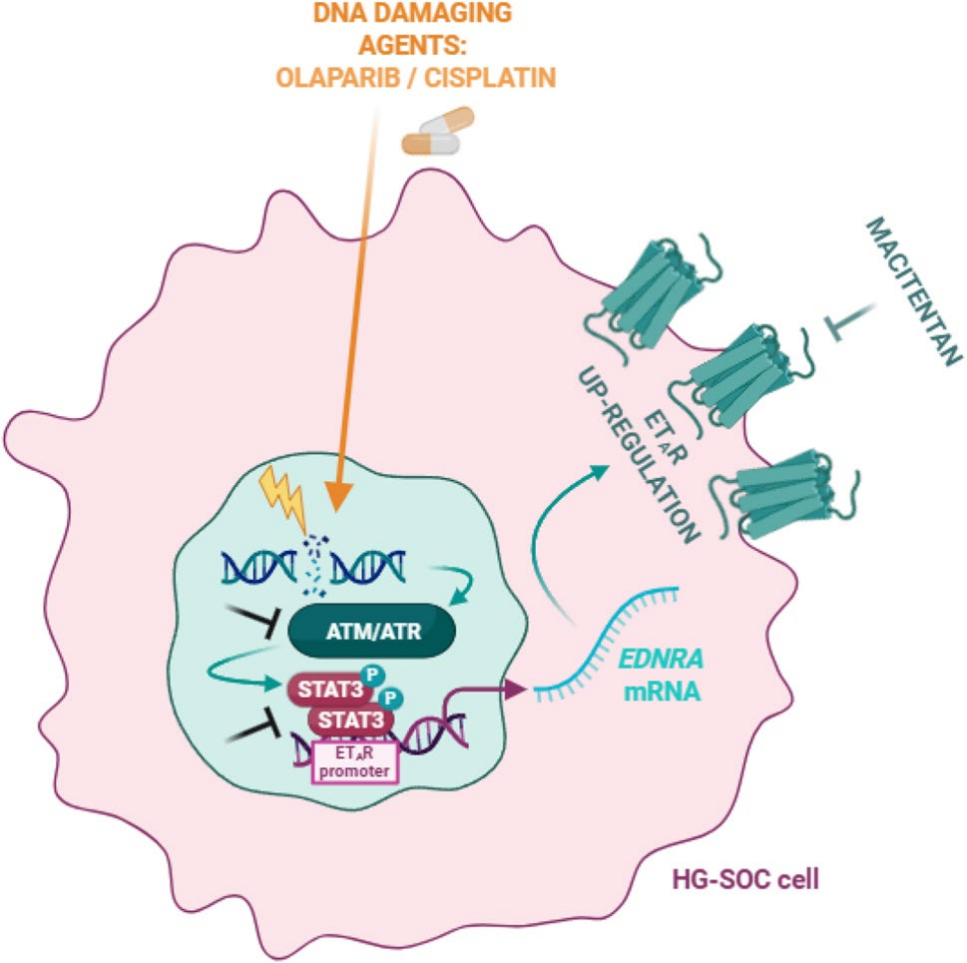
Working model illustrating how DNA damaging agents drive ET_A_R overexpression in HG-SOC. At molecular level, the activation of the DDR pathway triggers the STAT3 transcriptional machinery, upregulating ET_A_R expression. The co-administration of ATRi and PARPi, halting the ET_A_R-mediated drug adaptive response, enhances PARPi efficacy in HG-SOC cells. Further, the incorporation of macitentan to such therapeutic scenario potentiates ATRi and PARPi cytotoxic effects in HG-SOC

It is extensively recognized that the heterogeneous nature that characterizes the HG-SOC fuels the tumour cell adaptability to diverse conditions, including the selective pressure exerted by anti-cancer drugs, giving rise to an ineffective response to standard of care therapies, causing disease relapse in many patients [5, 6, 14]. Given the complexity and the variety of the processes supporting PARPi and platinum-based treatment failure [2, 4, 7], it is crucial to broaden the knowledge of those mechanisms that are decisive of an unfavourable response to these compounds, providing a starting point to overcome drug resistance in HG-SOC. In this regard, our study unveiled how PARPi and platinum-based therapies sustained a drug escape strategy by upregulating the expression of ET_A_R that protects HG-SOC cells from drug-induced apoptosis. From a molecular perspective, we uncovered a previously unrecognized mechanism whereby, in HG-SOC cells, DNA-damaging agents, regardless of *BRCA1/2* mutations, triggers a DDR-STAT3 interplay, that is in charge of the ET_A_R transcriptional induction. Importantly, our results shed light on how the addition of DDRi to the PARPi-mediated synthetic lethality effect, hindering the STAT3-driven ET_A_R transcription, prevents the ET_A_R-driven drug escape route. Of clinical significance, the incorporation of an ET_A_R targeting compound to DDRi + PARPi-based regimens, blocking an adaptive circuit, further potentiates the olaparib therapeutic effect, impeding the HG-SOC metastatic relapse (Fig. 8), providing a glimpse into the safeguard function of ET_A_R signalling in ovarian cancer.

Previous observations in HG-SOC patients showed that high ET_A_R expression predicted resistance to platinum-based therapy and poorer clinical outcomes [9–12], reinforcing the idea that dampening ET_A_R activity might improve the response to chemotherapy. Given the well-documented overlap between the mechanisms underlying platinum and PARPi resistance [38], we can hypothesize that high ET_A_R expression may hold prognostic value also within the context of the unfavourable response to PARPi. In support of this hypothesis, our data show that PARPi-unresposive cells, exhibiting a greater expression of ET_A_R compared to the parental counterpart, regain PARPi sensitivity upon ET_A_R depletion, suggesting that the aberrant activation of the ET_A_R-driven signalling may function as an escape route through which tumour cells evade PARPi therapy engaging an adaptive mechanism to survive.

Currently, there is no reported evidence addressing whether and how PARPi-based therapy or chemotherapy could modulate ET_A_R expression, highlighting a gap in our understanding about the molecular events underpinning ET_A_R overexpression associated to tumour cell resilience to these treatments in HG-SOC. Given that chemotherapy and PARPi are used as standard of care in HG-SOC, research studies focusing on the characterization of the expression of ET_A_R and ET_A_R-derived adaptive mechanisms are of critical importance in this tumour milieu.

We deepened this aspect, unveiling an ATM/ATR/STAT3 signalling networking in charge of ET_A_R overexpression upon DNA damage agent treatment, pinpointing potential targets for therapeutic exploitation. Our findings are in close agreement with recent studies delineating how in other tumour types the blockade of DDR players hampers the STAT3-dependent transcriptional program [39–41]. These observations could be an instrumental step to improve the efficacy of PARP inhibition in ovarian cancer [42–45] showing that, beyond modulating ATR/CHK1 or ATM/CHK2 [46–48], STAT3 exerts a role downstream of the olaparib-induced DDR pathway, regulating the transcription of ET_A_R, identified as key vulnerability in response to DNA damage.

Available clinical trials with PARPi in combination with ATRi have shown that only a subset of patients with specific genomic alterations shows improved responses, suggesting an urgent need for the identification of predictive biomarkers to underpin patient selection strategies for ATRi and PARPi-based therapies [30], together with a better definition of the intrinsic mechanism of PARPi unresponsiveness. Our results are in line with observations conducted in ovarian cancer cells poor responsive to PARPi, that demonstrate how PARPi + ATRi combination bring an increased accumulation of DNA double-strand breaks and replication fork collapse, ultimately leading to the increase of cell apoptosis [49–51].

Although ATR is crucial for repairing DNA damage caused by olaparib, we now propose that HG-SOC cells, expressing endogenous ET_A_R, are particularly sensitive to ATR inhibitor treatments because ATR inhibition counteracts the olaparib-induced transcription of ET_A_R. HG-SOC cells overexpressing ET_A_R are less prone to olaparib, suggesting that the ET_A_R expression level might predict patients’ tumour sensitivity to the combination of ATRi and PARPi treatment. In this scenario, recognizing ET_A_R as a potential prognostic marker within the context of HRP, we provide insight into the therapeutic intervention designed to target such particularly high-risk HG-SOC patient subgroup. In particular, our findings suggest a potential therapeutic approach by which HG-SOC patients highly expressing ET_A_R could benefit from the incorporation of the dual ET-1R antagonist macitentan into existing combination regimens, such as PARPi + ATRi. Such small molecule, approved for the treatment of the pulmonary arterial hypertension and known for its well-tolerated toxicity profile [52–54], and able to block simultaneously HG-SOC cell functions mediated by ET_A_R, and the tumour-supportive activity of stromal components expressing ET_B_R [14], may represent a very promising candidate for drug repurposing in HG-SOC patients, even in the setting of advanced and resistant forms of the disease. The ability of macitentan to hamper the tumour/stroma interactions, disrupting the interdependencies in the tumour ecosystem [14], provides the rational to use this drug in combined regimens to combat resistance.

With the ongoing development of novel inhibitors and the identification of ET_A_R as a rescue mechanism from olaparib-induced cell death, the combination of PARPi + ATRi with ET-1R therapeutics is likely to undergo further preclinical testing and future clinical studies for the treatment of highly aggressive and refractory HG-SOC. Furthermore, while our study focused on HG-SOC models, it might have a broader implication for diverse tumour types frequently expressing ET_A_R and DDR players, that can respond to PARPi therapies.

## Conclusions

Overall, these findings elucidate an intrinsic mechanism underlying olaparib and cisplatin unresponsiveness in HG-SOC, wherein ET_A_R overexpression has been ascribed as a vulnerability that can be exploited through the combined use of ET_A_R targeting compounds with PARPi and ATRi, positioning the ET_A_R-driven escape signalling as a determinant able to undermine HG-SOC patient favourable response to the currently available HG-SOC therapeutic solutions.

## Supplementary Information

Supplementary Material 1.

Supplementary Material 2.

Supplementary Material 3.

Supplementary Material 4.

## Data Availability

The datasets used and/or analyzed during the current study are available from the corresponding author on reasonable request.The RNA-Seq data have been deposited at the GEO repository database with the accession code GSE196065.

## Abbreviations

Ab: Antibody
ATM: Ataxia Telangiectasia Mutated
ATMi: ATM inhibitor
ATR: Ataxia Telangiectasia and Rad3-Related Protein
ATRi: ATR inhibitor
bp: Base pair
CHK1: Checkpoint kinase 1
CHK2: Checkpoint kinase 2
ChIP: Chromatin immunoprecipitation
CTR: Control
DDR: DNA Damage response
DDRi: DNA Damage response inhibitor
ET-1: Endothelin-1
ET-1R: Endothelin-1 receptor
ET_A_R: Endothelin A receptor
ET_B_R: Endothelin B receptor
GEO: Gene expression omnibus
GPCR: G protein coupled receptor
GSEA: Gene set enrichment analysis
HG-SOC: High-grade serous ovarian cancer
h: Hours
HR: Homologous recombination
HRD: Homologous recombination deficient
HRP: Homologous recombination proficient
IB: Immunoblotting
IF: Immunofluorescence
KEGG: Kyoto Encyclopedia of Genes and Genomes
MAC: Macitentan
NGS: Next generation sequencing
OS: Overall survival
PARP: Poly ADP-ribose polymerase
PARPi: PARP inhibitors
PD: Patient-derived
PDX: Patient-derived xenograft
PFS: Progression free survival
qRT-PCR: Quantitative reverse transcription polymerase chain reaction
RNA-Seq: RNA sequencing
SD: Standard deviation
TCGA: The Cancer Genome Atlas
TF: Transcription factor
