# Supplementary material for: DNA damaging agents boost the transcription of endothelin A receptor in high-grade serous ovarian cancer
Source: J Exp Clin Cancer Res. 2025 Dec 6;45:12. doi: 10.1186/s13046-025-03607-0 (PMC12784578; doi:10.1186/s13046-025-03607-0)
Supplement: Supplementary file 1 — Supplementary Material 1. [file 13046_2025_3607_MOESM1_ESM.pdf]

Additional File 1. Supplementary Figures

DNA damaging agents boost the transcription of endothelin A receptor in high-grade serous ovarian cancer

Celia Roman[1], Rosanna Sestito[1], Valentina Caprara[1], Andrea Sacconi[2], Giovanni Blandino[2], Anna Bagnato[1*] and Piera Tocci[1*]

[1]Preclinical Models and New Therapeutic Agents Unit, Istituto di Ricovero e Cura a Carattere Scientifico (IRCCS), Regina Elena National Cancer Institute, Rome, Italy; [2]Translational Oncology Research Unit, IRCCS, Regina Elena National Cancer Institute, Rome, Italy.

*Corresponding authors: Piera Tocci (Tel: +39 06522570; E-mail: piera.tocci@ifo.it); Anna Bagnato (Tel: +39 0652665618; E-mail: annateresa.bagnato@ifo.it).

This document contains:

Supplementary Figures S1-4 and related figure legends

A

	p53	BRCA1	BRCA2	HR status
PD HG-SOC	R337T C>G			HRP
OVCAR 8	376-1G>A	Promoter hypermethylation		HRD
KURAMOCHI	D281Y		Mutation	HRD

Promoter hypermethylation
Mutation

B

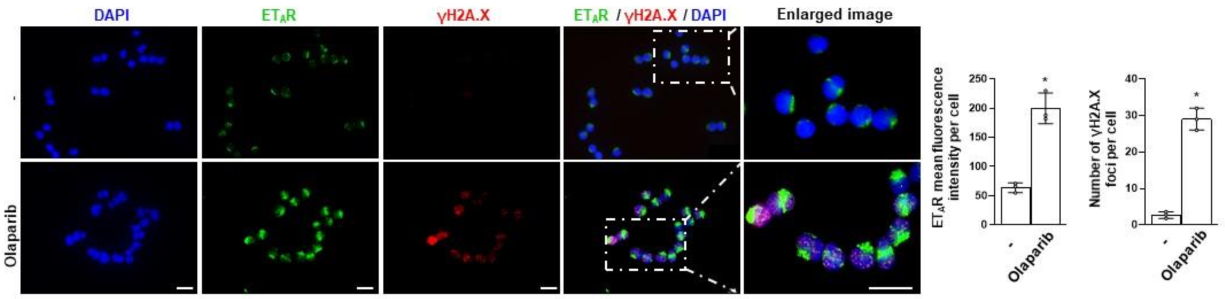

Supplementary Fig. S1. Olaparib induces the overexpression of endothelin A receptor. **A** Mutational landscape in high-grade serous ovarian cancer (HG-SOC) cells. Table showing p53, BRCA1 and BRCA2 gene mutations and the homologous recombination status in patient-derived (PD HG-SOC), OVCAR8 and Kuramochi cells. HRP (homologous recombination proficient); HRD (homologous recombination deficient). **B** Representative images from immunofluorescence (IF) analysis of ET_AR and γH2A.X of PD HG-SOC cells treated or not with 10 µM olaparib for 48 h. Scale bar: 20 µm. Right graphs show the mean fluorescence intensity of ET_AR immunostained PD HG-SOC cells and the number of γH2A.X foci per cell, represented as mean ± SD. An average of 200 cells per condition was analysed (n=3, *p < 0,003 vs non-treated cells).

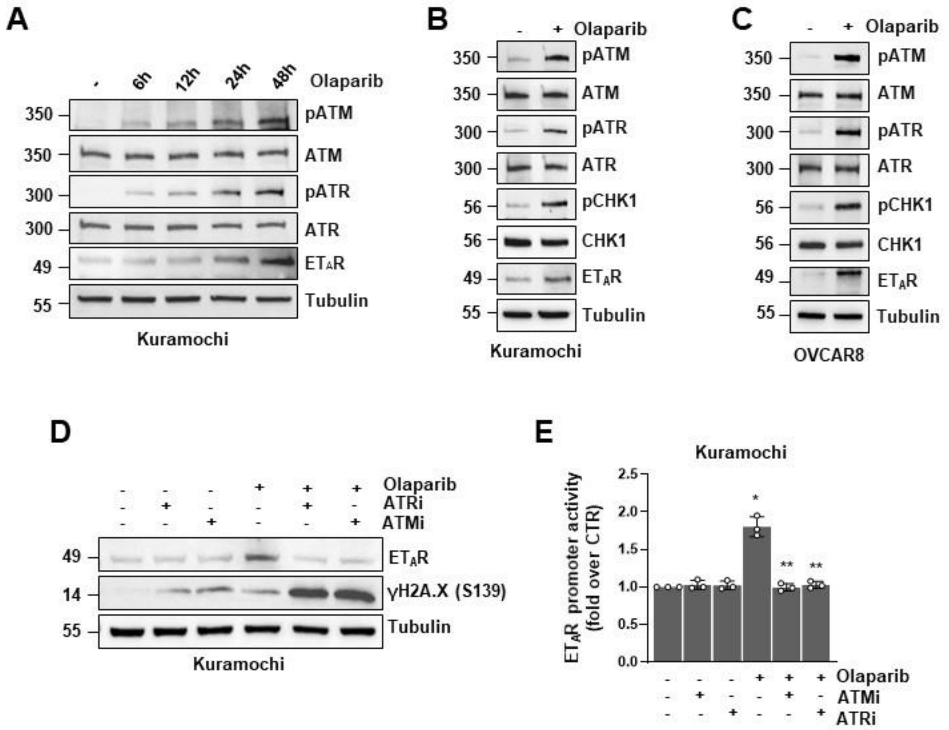

Supplementary Fig. S2. Olaparib-induced activation of the DNA Damage Response cascade mediates ET$_A$R upregulation. **A** Immunoblotting (IB) of pATM, ATM, pATR, ATR and ET$_A$R protein expression levels of Kuramochi cells treated or not with 10 μM olaparib for the indicated times. Tubulin was used as loading control. **B, C** IB analyses detecting pATM, ATM, pATR, ATR, pCHK1, CHK1 and ET$_A$R expression levels of Kuramochi (B) or OVCAR8 (C) cells treated or not with 10 μM olaparib for 48 h. **D** IB of ET$_A$R and γH2A.X protein expression levels of Kuramochi cells treated with olaparib (10 μM) and/or ATRi (2,5 μM) or ATMi (5 μM). **E** ET$_A$R promoter activity (fold over CTR) of Kuramochi cells treated as in *D* and transfected with an ET$_A$R promoter reporter plasmid for 48 h. Data are presented as mean ± SD (n=3, *p < 0,01 vs CTR; **p < 0,06 vs olaparib-treated cells).

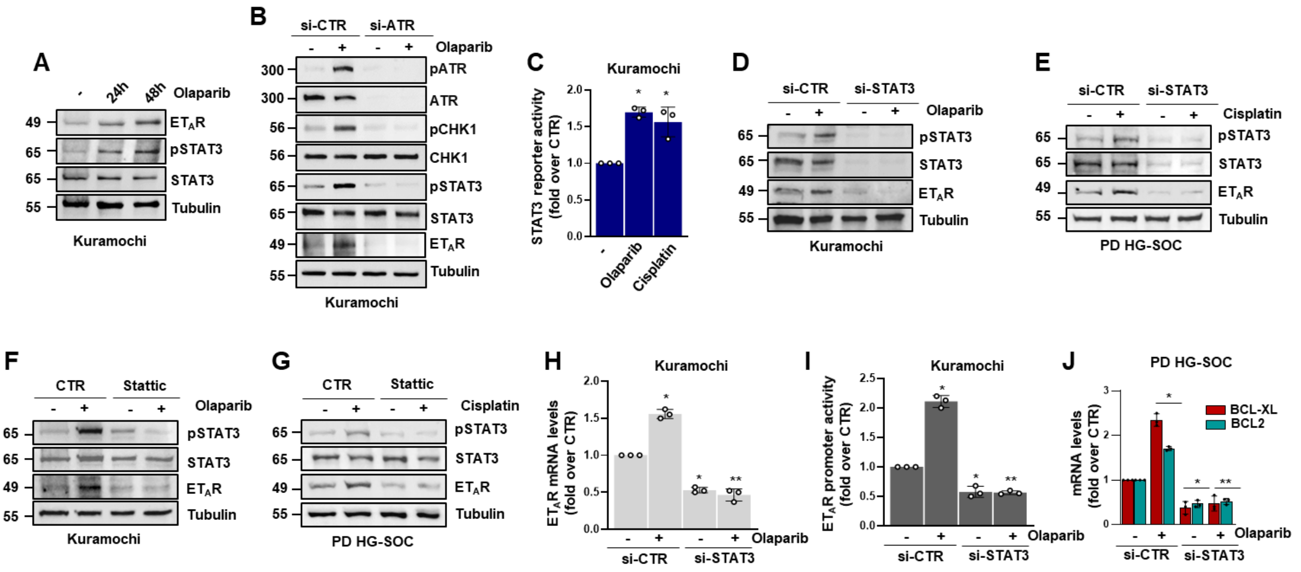

Supplementary Fig. S3. STAT3 transcriptional activity is required for the olaparib-induced ET$_A$R upregulation. **A** IB of ET$_A$R, pSTAT3 and STAT3 protein expression levels from total extracts of Kuramochi cells treated with 10 µM olaparib for 24 h or 48 h. Tubulin was used as loading control. **B** IB of pATR, ATR, pCHK1, CHK1, pSTAT3, STAT3 and ET$_A$R protein expression levels of Kuramochi cells transfected with si-CTR or si-ATR for 72 h and treated or not with olaparib for 48 h. **C** Luciferase activity (fold over CTR) detected in Kuramochi cells transfected with a STAT3 reporter vector and treated or not with 10 µM olaparib or cisplatin for 48 h. Data are presented as mean ± SD (n=3, *p < 0,05 vs non-treated cells). **D** IB of pSTAT3, STAT3 and ET$_A$R expression levels of Kuramochi cells transfected with si-CTR or si-STAT3 for 72 h and treated or not with olaparib for 48 h. **E** pSTAT3, STAT3 and ET$_A$R protein expression levels evaluated by IB in PD HG-SOC cells transfected with si-CTR or si-STAT3 for 72 h and treated or not with 10 µM cisplatin for 48 h. **F** IB of pSTAT3, STAT3 and ET$_A$R expression levels of Kuramochi cells treated with stattic (10 µM) and/or olaparib (10 µM) for 48 h. **G** IB of pSTAT3, STAT3 and ET$_A$R protein expression levels of PD HG-SOC cells treated with stattic (10 µM) and/or cisplatin (10 µM) for 48 h. **H** qRT-PCR analysis of ET$_A$R mRNA expression levels (fold over CTR) in Kuramochi cells transfected with si-CTR or si-STAT3 for 72 h and treated or not with olaparib for 48 h. Data are presented as mean ± SD (n=3, *p < 0,004 vs si-CTR non-treated cells; **p < 0,0001 vs olaparib-treated cells). **I** ET$_A$R

promoter activity (fold over CTR) of Kuramochi cells treated and transfected as in *H* and with an ET$_A$R promoter reporter plasmid for 48 h. Data are presented as mean ± SD (n=3, *p < 0,02 vs si-CTR non-treated cells; **p < 0,0007 vs olaparib-treated cells). **J** qRT-PCR analysis of BCL-XL and BCL2 mRNA expression levels (fold over CTR) in PD HG-SOC cells transfected with si-CTR or si-STAT3 for 72 h and treated or not with olaparib for 48 h (n=3, *p < 0,02 vs si-CTR non-treated cells; **p < 0,0001 vs olaparib-treated cells).

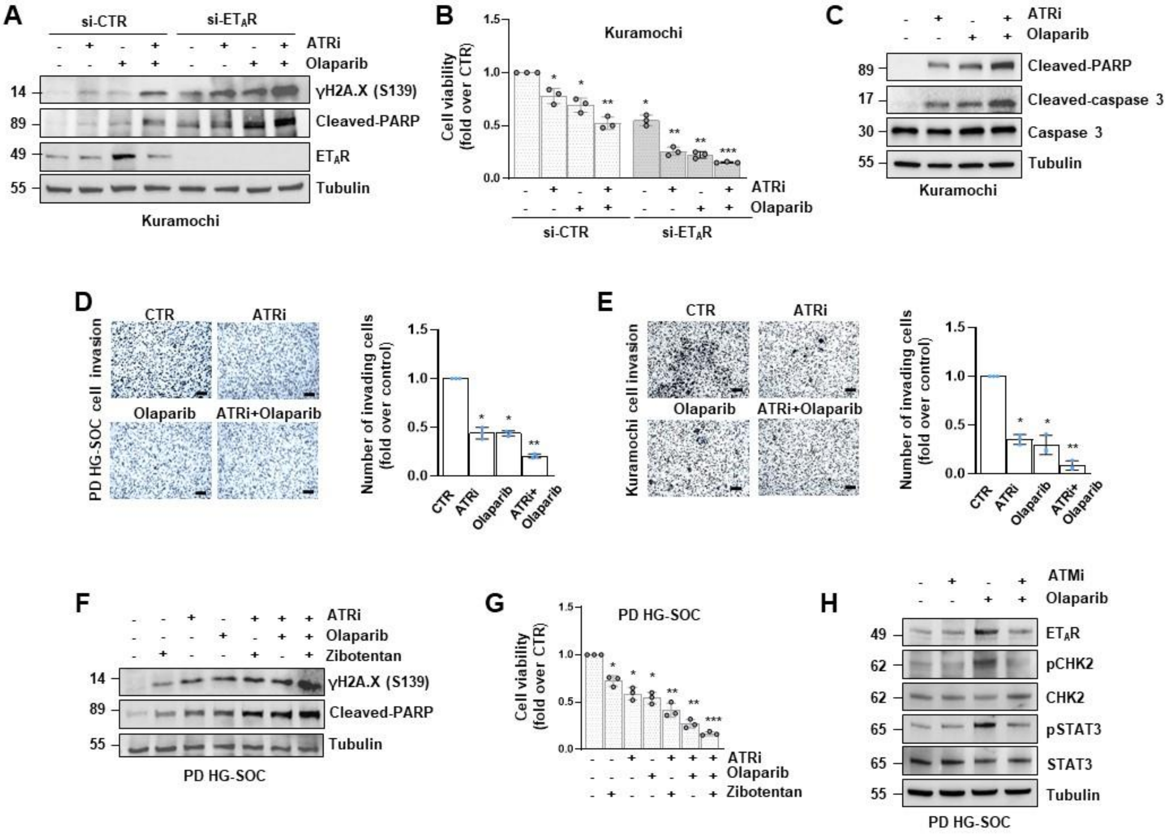

Supplementary Fig. S4. Combining olaparib with ET$_A$R/DDR inhibition potentiates DNA damage and apoptosis. **A** IB of γH2A.X, cleaved-PARP and ET$_A$R protein expression levels of Kuramochi cells transfected with si-CTR or si-ET$_A$R for 72 h and treated or not with ATRi (2,5 µM) and/or olaparib (10 µM) for 48 h. **B** Cell viability of Kuramochi cells treated and transfected as in *A*. Relative luminescence units were read as an indicator of viability and displayed as mean ± SD, expressed as fold over CTR (n=3; *p < 0,04 vs si-CTR non-treated cells; **p < 0,04 vs si-CTR ATRi- or olaparib-treated cells; ***p < 0,008 vs si-CTR ATRi + olaparib-treated cells). **C** IB of cleaved-PARP, cleaved-

caspase 3 and total caspase 3 protein expression levels of Kuramochi cells treated with ATRi and/or olaparib for 48 h. **D, E** Representative images of the invasion assay with PD HG-SOC (D) or Kuramochi (E) cells treated with ATRi and/or olaparib for 48 h, scale bar: 100 µm. The right graphs show the number of invading cells represented as mean ± SD, expressed as fold over CTR (n=3; *p < 0,004 vs PD HG-SOC non-treated cells; **p < 0,0005 vs PD HG-SOC olaparib-treated cells; n=3; *p < 0,007 vs Kuramochi non-treated cells; **p < 0,05 vs Kuramochi olaparib-treated cells). **F** IB of γH2A.X and cleaved-PARP protein expression levels of PD HG-SOC cells treated with ATRi, olaparib and/or zibotentan (1 µM) for 48 h. **G** Cell viability in PD HG-SOC cells treated as in *F* (n=3; *p < 0,02 vs non-treated cells; **p < 0,007 vs zibotentan- or olaparib-treated cells; ***p < 0,05 vs ATRi + olaparib-treated cells). **H** IB of ET_AR, pSTAT3, STAT3, pCHK2 and CHK2 protein expression levels of PD HG-SOC cells treated with olaparib (10 µM) and/or ATMi (5 µM) for 48 h.
