# Supplementary material for: DNA damaging agents boost the transcription of endothelin A receptor in high-grade serous ovarian cancer
Source: J Exp Clin Cancer Res. 2025 Dec 6;45:12. doi: 10.1186/s13046-025-03607-0 (PMC12784578; doi:10.1186/s13046-025-03607-0)

Uncropped Figure 2

Figure 2B

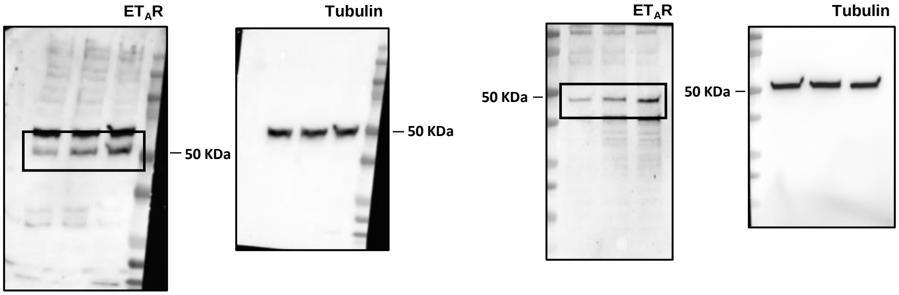

Figure 2C

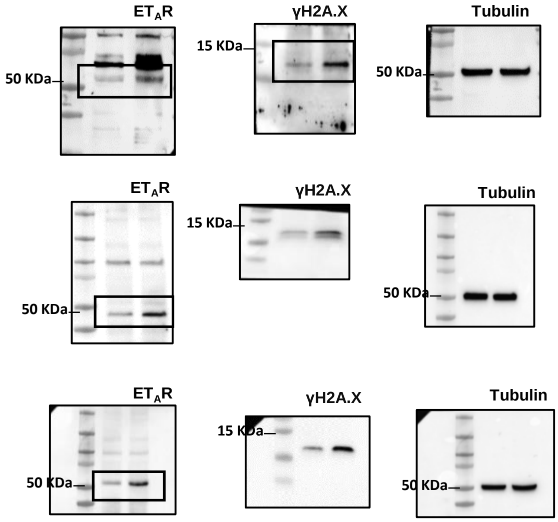

Figure 2F

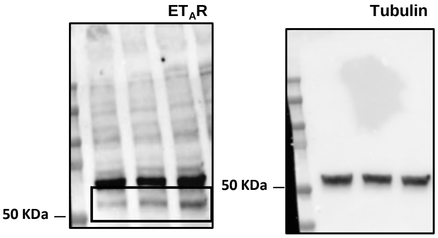

Figure 2H

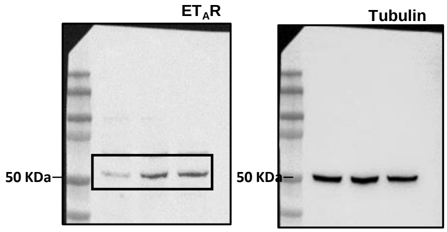

Uncropped Figure 3

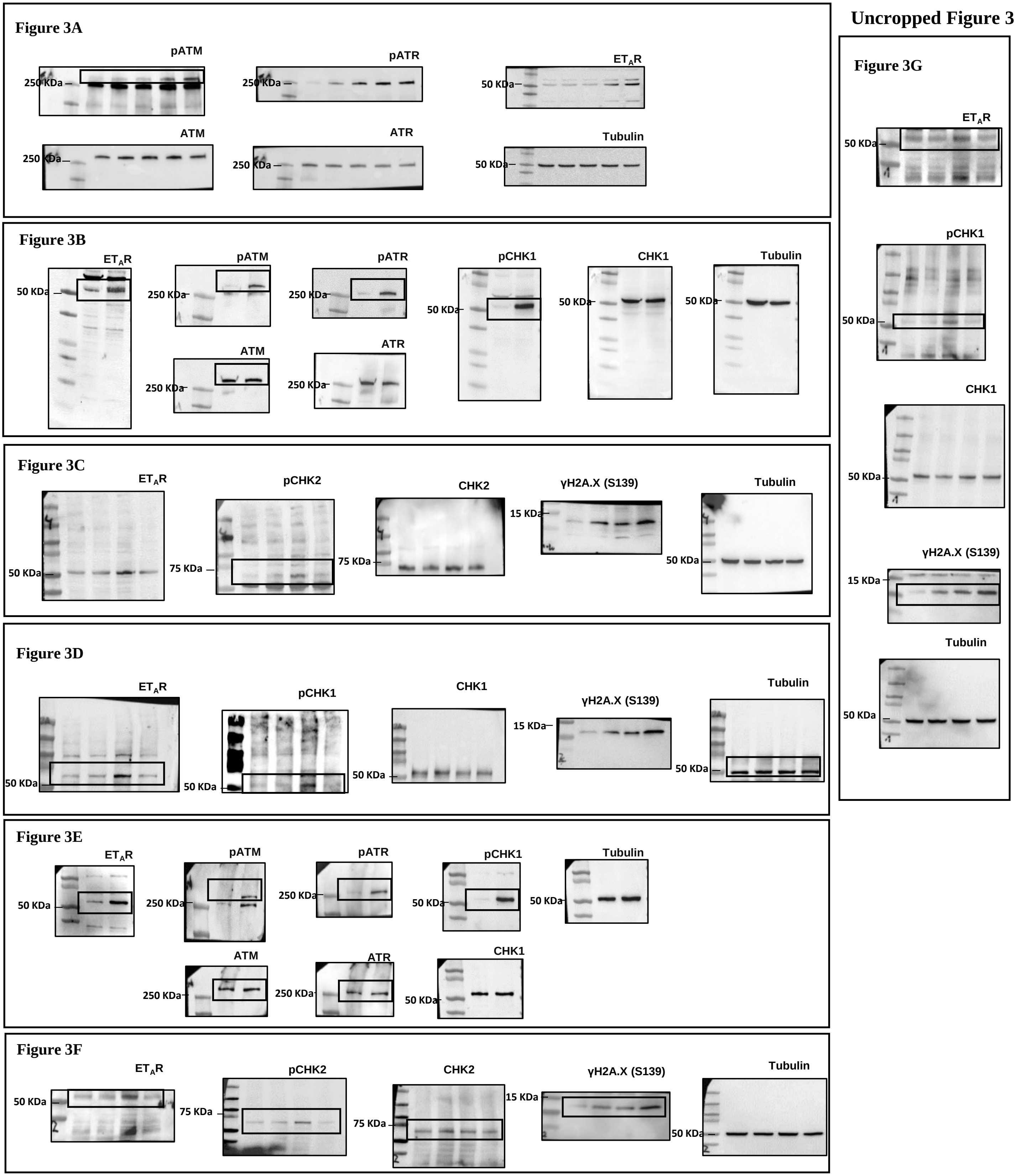

Uncropped Figure 5

Figure 5C

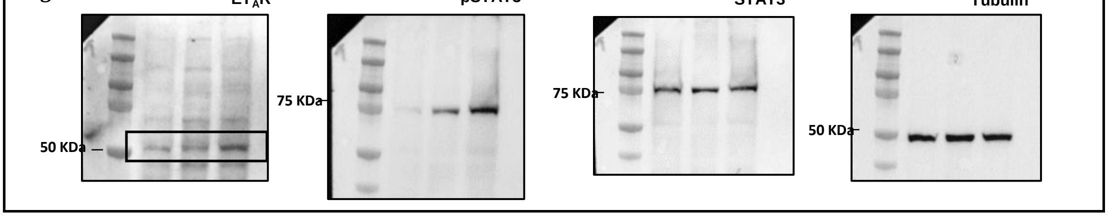

Figure 5D

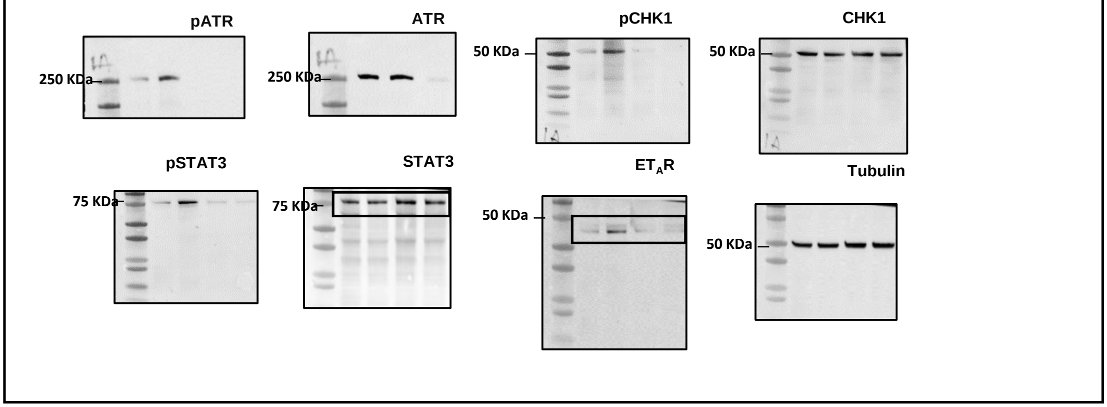

Figure 5H

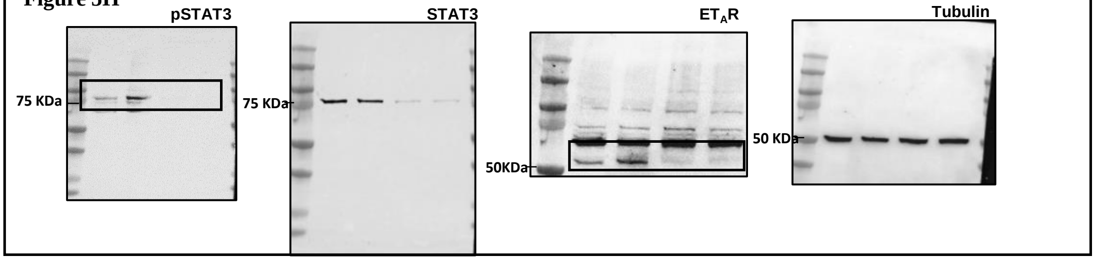

Figure 5I

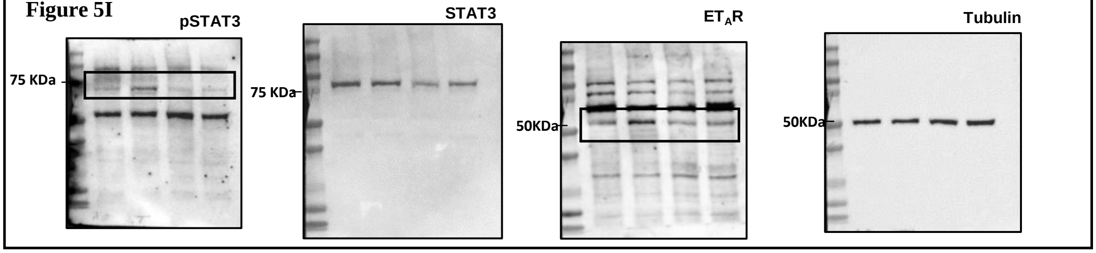

Figure 5M

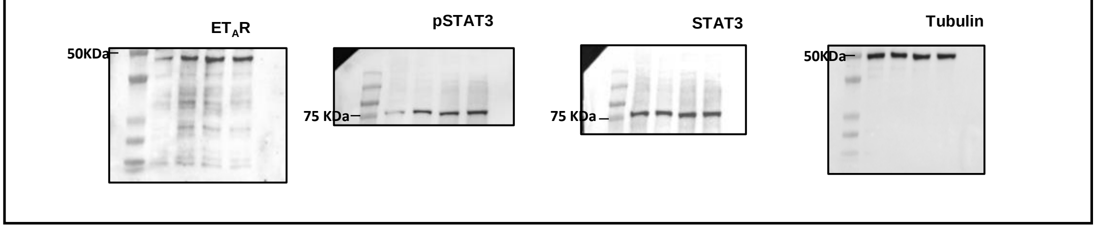

Uncropped Figure 6

Figure 6B

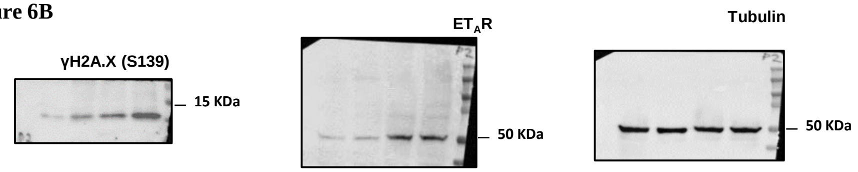

Figure 6C

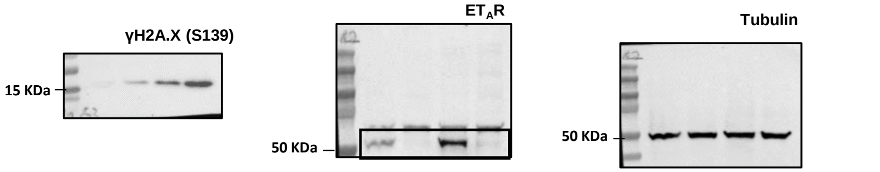

Figure 6D

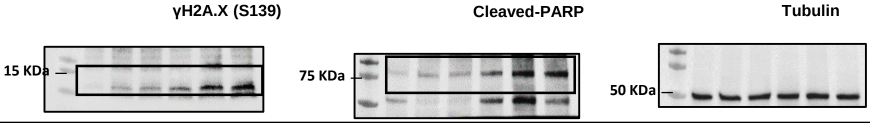

Figure 6F

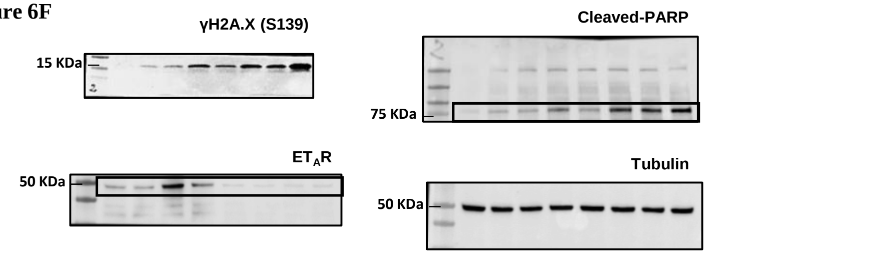

Figure 6I

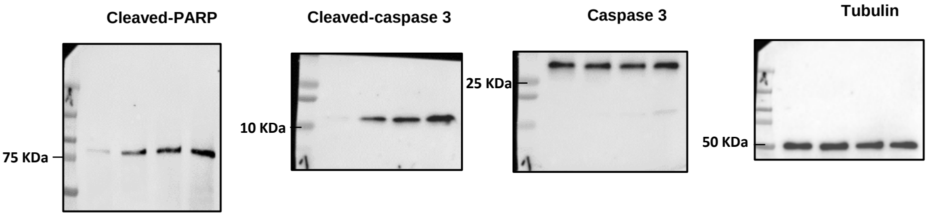

Figure 6J

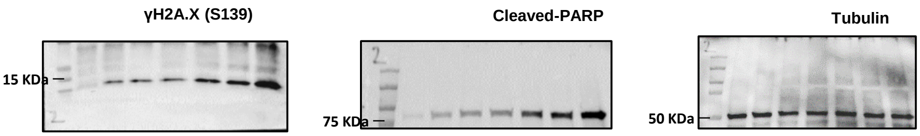

Figure 6L

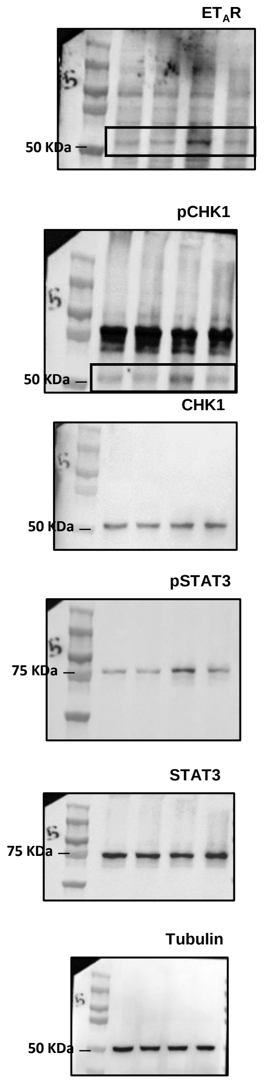

Uncropped Figure 7

Figure 7E

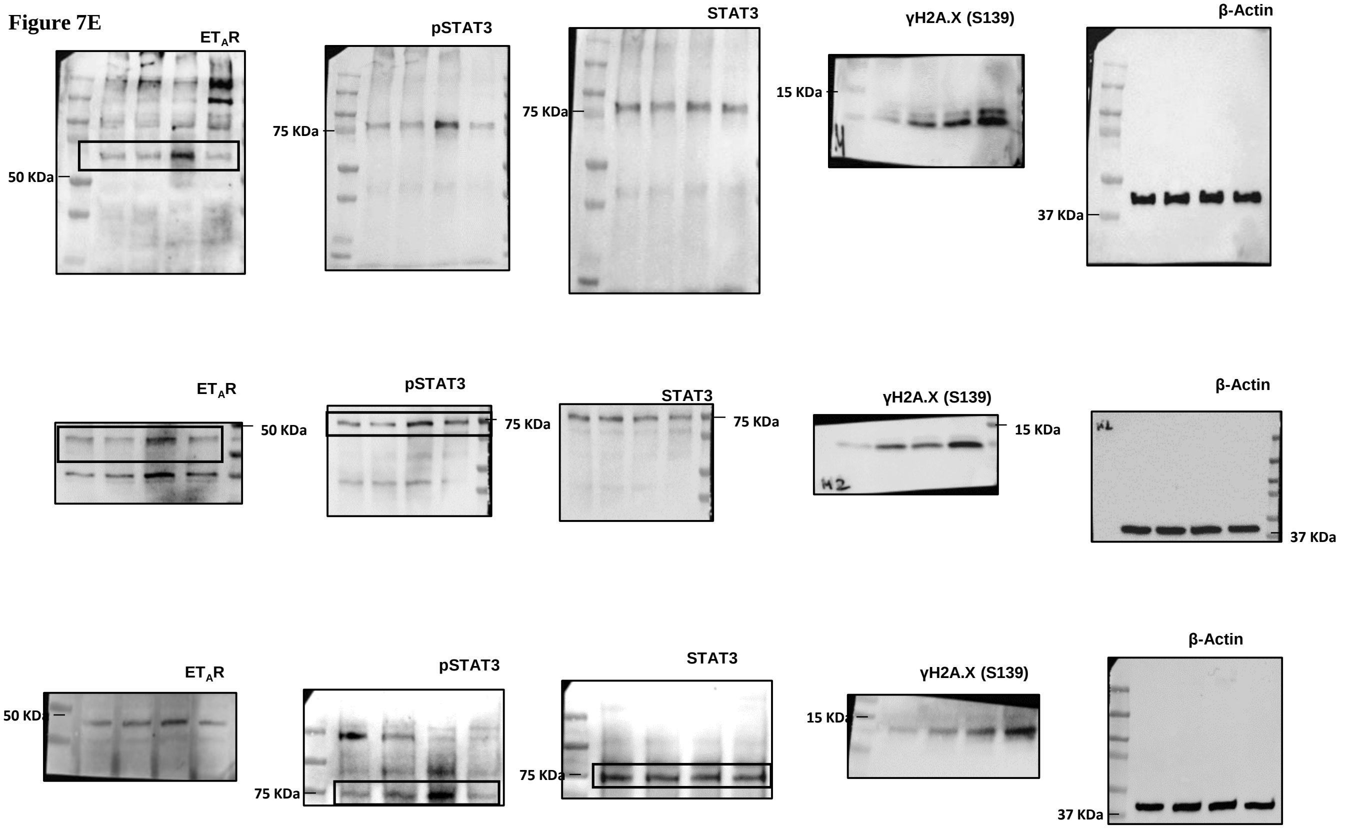

Figure 7I

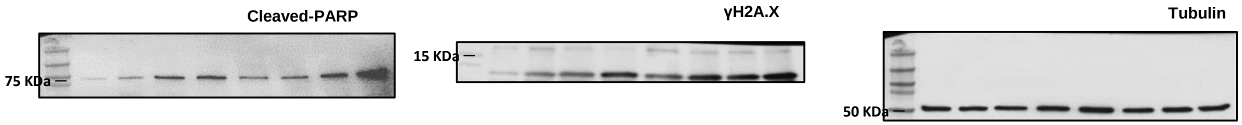

Supplement: Supplementary file 4 — Supplementary Material 4. [file 13046_2025_3607_MOESM4_ESM.pdf]
